# Pan-transcriptome reveals a large accessory genome contribution to gene expression variation in yeast

**DOI:** 10.1038/s41588-024-01769-9

**Published:** 2024-05-22

**Authors:** Élodie Caudal, Victor Loegler, Fabien Dutreux, Nikolaos Vakirlis, Élie Teyssonnière, Claudia Caradec, Anne Friedrich, Jing Hou, Joseph Schacherer

**Affiliations:** 1 Université de Strasbourg, CNRS GMGM UMR 7156, Strasbourg, France; 2 Institut Universitaire de France (IUF), Paris, France

**Keywords:** Gene expression, Gene regulation

## Abstract

Gene expression is an essential step in the translation of genotypes into phenotypes. However, little is known about the transcriptome architecture and the underlying genetic effects at the species level. Here we generated and analyzed the pan-transcriptome of ~1,000 yeast natural isolates across 4,977 core and 1,468 accessory genes. We found that the accessory genome is an underappreciated driver of transcriptome divergence. Global gene expression patterns combined with population structure showed that variation in heritable expression mainly lies within subpopulation-specific signatures, for which accessory genes are overrepresented. Genome-wide association analyses consistently highlighted that accessory genes are associated with proportionally more variants with larger effect sizes, illustrating the critical role of the accessory genome on the transcriptional landscape within and between populations.

## Abstract (web summary)

Insight from the transcriptomes of 1,032 *Saccharomyces cerevisiae* natural isolates emphasizes the essential contribution of accessory genes to the species-level transcriptional landscape.

## Main

Gene expression is the primary step of a process by which information encoded in the genome is converted into traits. Genetic variants affecting gene expression contribute to phenotypic diversity^1–4^. Dissection of the genetic regulation of different molecular intermediates leading to the final phenotypes is therefore essential for understanding many aspects of biology. Genetic variants or loci associated with gene expression variation (that is, expression quantitative trait loci (eQTLs)) have been identified using linkage and genome-wide association mappings in several organisms, uncovering the general mechanisms of transcription regulation^5–16^. Genetic variants underlying variation in gene expression can be located close or distant to the affected gene and are hence considered as either *cis*-acting (local eQTLs) or *trans*-acting (distant eQTLs), respectively. Studies highlighted the fact that local eQTLs have larger effects on gene expression than distant eQTLs^5–7^. However, a gene is often regulated by multiple distant eQTLs and this set of *trans*-regulatory variants tend to explain a larger fraction of the variance overall than its *cis*-eQTLs^6^.

In recent years, large-scale transcriptomic surveys were carried out in multiple model and non-model systems^5,7,12,14,17^, most notably the Genotype-Tissue Expression project in humans, which covers gene expression data across 49 tissues from up to 838 individuals^12^. These studies were extremely insightful regarding the regulation of tissue-specific gene expression and extensively cataloged *cis*-acting variants acting across tissues^12^. However, because of the large genomes and relatively limited sample sizes, *trans-*eQTLs remained difficult to detect in such settings, leaving part of the variance in gene expression still unexplained^12^. Moreover, most population-level studies focused on only a small fraction of genetic diversity, disregarding the wide variation in genetic content, namely the entire pangenome, and more precisely, the accessory genome. Finally, many species, including humans, display clear population structures that are often linked to the demographic, ecological and evolutionary histories of the subpopulations^17–19^. However, because of sampling limitations, the impact of the subpopulation structure on gene expression remains unclear; thus, no unified view of the regulation patterns of gene expression within and among populations is currently available.

Understanding the variation in patterns of gene expression at a population scale remains a challenge, but it should provide deeper insights into the molecular basis of phenotypic diversity and transcriptional network architecture. In this study, we took advantage of a population of 1,011 *Saccharomyces cerevisiae* yeast isolates we previously completely sequenced to explore the transcriptomic landscape at a population scale^20^. The *S. cerevisiae* yeast is a key model for investigating how genetic variants influence gene expression^6,7,21^. This species is characterized by a complex population structure with domesticated and wild subpopulations, and presents high genetic diversity^20^. Large-scale genome analysis of the 1,011 natural isolates also provided a pangenome definition of the species and a comprehensive view of genome variation at different levels, including copy number variants (CNVs) and gene content variation^20^. Finally, this large sample increased the power of genome-wide association studies (GWAS), allowing for in-depth characterization of local and distant regulatory variants that impact variation in gene expression. Together, these two genomic and transcriptomic datasets led to the most comprehensive insight into the regulation of genome-wide expression across species, which presently would be a challenge to achieve at this scale and at the same level of accuracy for other organisms. Our study advances the understanding of the genetic and functional architecture of the transcriptional landscape and its heritability at a species-wide scale.

## Results

### Pan-transcriptome dataset across 969 natural isolates

**Fig. 1:**
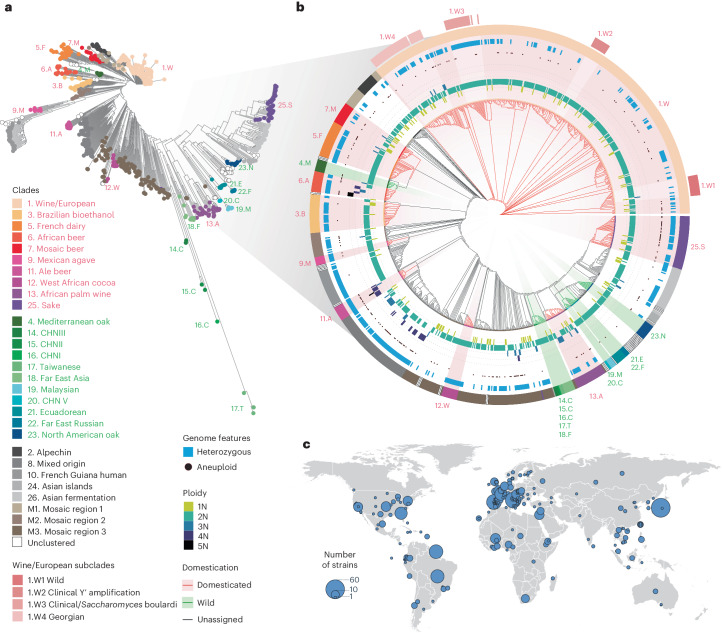
Origin and genomic diversity of 969 isolates. **a**, Neighbor-joining tree based on biallelic SNPs among 969 isolates included in our data. Previously defined subpopulations^20^ are color-coded. **b**, Detailed descriptions of several genomic features of the isolates. Circular cladogram for the 969 isolates; the colored branches correspond to domesticated (red) and wild (green) clusters; the ploidy levels for each isolate ranged from 1N to 5N; the presence of any aneuploidy is indicated by a black dot; heterozygosity is shown as a blue bar; clades and subclades are color-coded. **c**, Geographical distribution of the 969 isolates. The size of the circles indicates the number of isolates included from a given geographical location. The outline of the map was generated using the open-source R package ‘maps’ (v.3.4.2).

To gain a comprehensive overview of variation in gene expression at the species level, we performed RNA sequencing (RNA-seq) of a collection of 1,032 *S. cerevisiae* natural isolates^20,22^ and obtained 969 high-quality transcriptomes with at least 1 million mapped reads (Fig. 1, Supplementary Fig. 1a and Supplementary Table 1). We performed independent culture replicates for 29 samples (Supplementary Fig. 1b). The data are highly reproducible, with an average correlation of 0.94 between replicates (Supplementary Fig. 1c) and robust to different sample batches (Supplementary Fig. 1d). The genomes of all isolates were previously completely sequenced and extensively characterized, reflecting the broad genetic diversity of the species in terms of SNPs, gene CNVs, genome content variation (for example, introgressions, horizontal gene transfers), and aneuploidy-level and ploidy-level variation (Fig. 1a,b). We observed widespread dosage compensation on aneuploid chromosomes, with both gain-of-copy and loss-of-copy aneuploidies having a more similar expression level to euploid chromosomes than expected based on chromosome copy numbers (Supplementary Fig. 2). This dosage compensation effect is consistent with a previous study focusing on a smaller number of natural yeast isolates^23^.

**Fig. 2:**
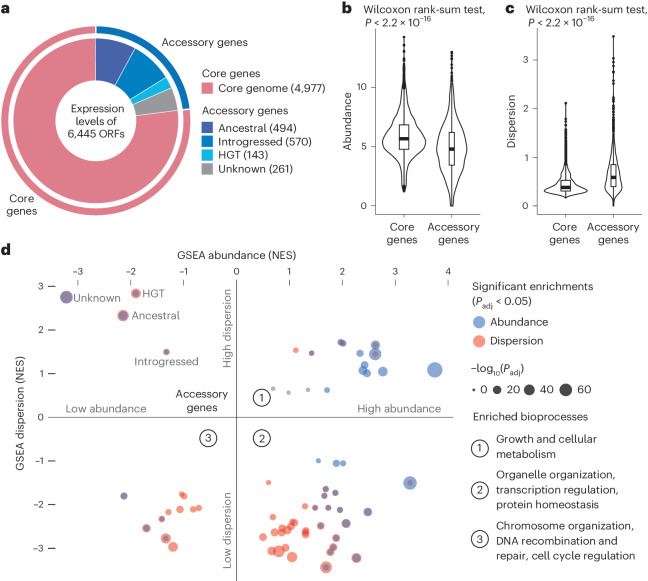
Functional description of the dataset. **a**, Number and distribution of all transcripts analyzed in the data, including 4,977 core genes and 1,468 accessory genes as previously annotated based on their genomes^20^. **b**,**c**, Global comparison of mean gene expression abundance (**b**) and dispersion (**c**) between core and accessory genes. Mean expression abundance was calculated as the mean log_2_ of the normalized read counts (transcripts per million (TPM)) across isolates for which the gene was present in their genome. For gene dispersion, we used mean absolute deviation (MAD), which is more robust to outliers and does not assume normality of expression levels compared with the s.d. For accessory genes, isolates that did not carry the given gene were excluded from the calculations. A two-sided Wilcoxon signed-rank test was performed on 4,977 core genes versus 1,468 accessory genes. The middle bar of the box plots corresponds to the median; the upper and lower bounds correspond to the third and first quartiles, respectively. The whiskers correspond to the upper and lower bounds 1.5 times the interquartile range (IQR) (Supplementary Fig. 3). **d**, GSEA results for expression abundance (*y* axis) and dispersion (*x* axis), presented as normalized enrichment scores (NES). Sixty-two non-redundant GO Slim biological process terms and four accessory gene subcategories were included. Significant enrichments are shown in blue (for abundance) and red (for dispersion). The summary terms for each quadrant are as indicated on the plot. Detailed distributions and enrichments for each term are presented in Supplementary Fig. 4.

The final set of 969 isolates were distributed across 26 well-defined clades that captured the ecological and geographical diversity of species, including several domesticated and wild subpopulations (Fig. 1a–c). Based the previously determined yeast pangenome^20^, we obtained the expression levels for 6,445 transcripts, including 4,977 core open reading frames (ORFs) and 1,468 accessory ORFs variably present across isolates (Fig. 2a, Supplementary Table 2 and 1002 Yeast Genome Project, datafile 1 (details on datafiles in the ʻData availabilityʼ section)).

### Accessory genes display unique transcriptional behavior

Each of the 6,445 genes in our dataset can be characterized by its overall expression level and its variation across the 969 isolates. To explore the global gene expression behavior, we defined two metrics, that is, abundance, which corresponds to the average expression level for a given gene across samples, and dispersion, which describes the variance across samples (Methods). By looking at the pangenome, we found that core and accessory genes display distinct patterns, which are characterized by a significantly lower abundance and higher dispersion of accessory gene expression compared with core genes (Fig. 2b,c). This difference is not biased by the stochastic variation associated with low levels of gene expression (Supplementary Fig. 3). From a functional perspective, we examined 62 broad and non-redundant Gene Ontology (GO) Slim terms related to biological processes using gene set enrichment analysis (GSEA) based on the rankings of mean expression abundance and dispersion across all genes. Among the 62 GO Slim terms, 59 were significantly enriched (false discovery rate (FDR) < 0.05) for abundance or dispersion, and were then grouped into three quadrants depending on the direction of enrichment (Fig. 2d and Supplementary Table 3). Specifically, genes involved in GO terms related to growth and cellular metabolism showed high abundance and high dispersion. By contrast, genes involved in GO terms related to organelle organization, transcription regulation and protein homeostasis showed high abundance but low dispersion. The low abundance and low dispersion quadrant was characteristic for genes involved in GO terms related to chromosome organization, DNA recombination and repair, and cell cycle regulation.

The added dimension of expression dispersion allowed us to distinguish sets of genes that most probably drove transcriptional variation in a population. For example, genes involved in metabolism and growth were among the most highly abundant and dispersed biological processes, which could reflect the diverse metabolic states and preferences across different isolates. Remarkably, unlike genes in all known major biological processes, accessory genes uniquely occupied the low-abundance, high-dispersion space (Fig. 2d and Supplementary Fig. 4). These patterns suggested that accessory genes probably represent a previously undercharacterized and unknown driver of transcriptional landscape diversity across species.

### Population-wide coexpression patterns recapitulate cellular functions

**Fig. 3:**
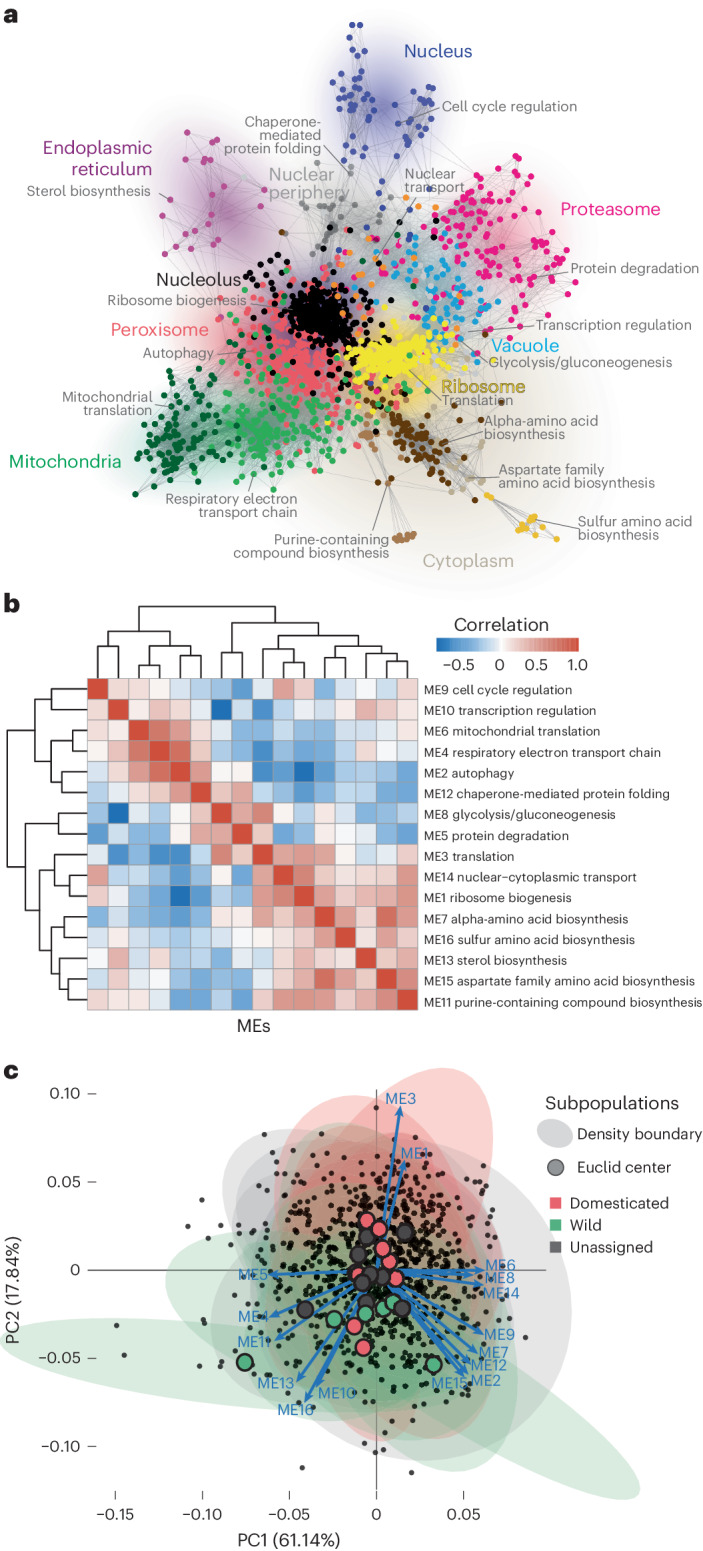
Whole-population-level gene coexpression network. **a**, Coexpression network based on pairwise gene expression profile similarities across 969 isolates. Nodes are colored according to the 16 coexpression modules detected using WGCNA. Modules were annotated according to GO term enrichment related to biological processes and are shown in gray. Enriched cellular compartments are annotated and indicated by the colored shading. **b**, Pairwise similarity matrix based on eigengene expression across the 16 modules. Modules were numbered in descending order of their sizes. **c**, PCA based on ME expression across isolates. The first two PCs were plotted. Density boundaries were drawn for each subpopulation and colored according to the domesticated, wild and unassigned clade annotations. Euclid centers for each subpopulation-based density boundary are as shown.

To explore the coordination of gene expression of different cellular processes in genetically distinct isolates, we constructed a species-wide gene coexpression network based on pairwise expression profile similarities across the population (Fig. 3a). Edges connected genes with similar expression profiles (Pearson’s *r* > 0.67); genes with fewer than five edges were excluded. The resulting network consisted of 1,797 genes displaying a scale-free architecture with clear modular topology (Fig. 3a). Using weighted correlation network analysis (WGCNA), we identified 16 coexpression modules localized to distinct regions of the network (Fig. 3a and Supplementary Table 4). Each module was enriched for a unique set of GO terms related to a similar biological process (Supplementary Table 5), with the largest module (432 genes) enriched for ribosome biogenesis, and the smallest module (13 genes) corresponding to genes involved in sulfur amino acid biosynthesis. The relative positions of modules in the network also reflected broader functional relationships and shared cellular localizations (Fig. 3a and Supplementary Table 5). This hierarchical organization was further illustrated by examining the pairwise correlations between module eigengenes (ME), which showed that modules involved in distinct but related biological processes were clustered together (Fig. 3b). Therefore, these coexpression patterns recapitulate the network of cellular functions.

We next focused on potential coexpression signatures related to the structure of the subpopulations. We performed principal component analysis (PCA) using eigengene expressions across all 16 identified modules (Fig. 3c). Globally, there was no clear delineation among different subpopulations based on the first two principal components (PCs) (Fig. 3c). This lack of strong subpopulation impact was corroborated by the general absence of differential subpopulation-specific coexpression (Supplementary Fig. 5), indicating that the coexpression network was robust to genetic variation across the population.

Overall, this population-wide coexpression network captured the topological organization of the cell by displaying the hierarchical relationships between functionally defined modules. The network was globally robust to the population structure, highlighting the part of the transcriptional landscape that was coordinated, hierarchical and functionally conserved at the species level.

### Subpopulation-specific signatures related to domestication

To identify subpopulation-specific transcriptional signatures, we performed differential gene expression analyses by comparing each clade to the rest of the population (Methods). We filtered out genes for which expression was not detected in over half of the population, resulting in an input set of 6,116 genes. We found 2,209 unique differentially expressed genes (DEGs) across clades (Supplementary Table 6). The number of significant DEGs detected in a given clade did not necessarily correlate with the size of the subpopulation (Supplementary Fig. 6a and Supplementary Table 4). On average, each subpopulation showed ~130 DEGs, ranging from 390 for the French dairy clade (5.F, 30 isolates) to zero for the CHNII clade (15.C, two isolates) (Supplementary Fig. 6a and Supplementary Table 4).

**Fig. 4:**
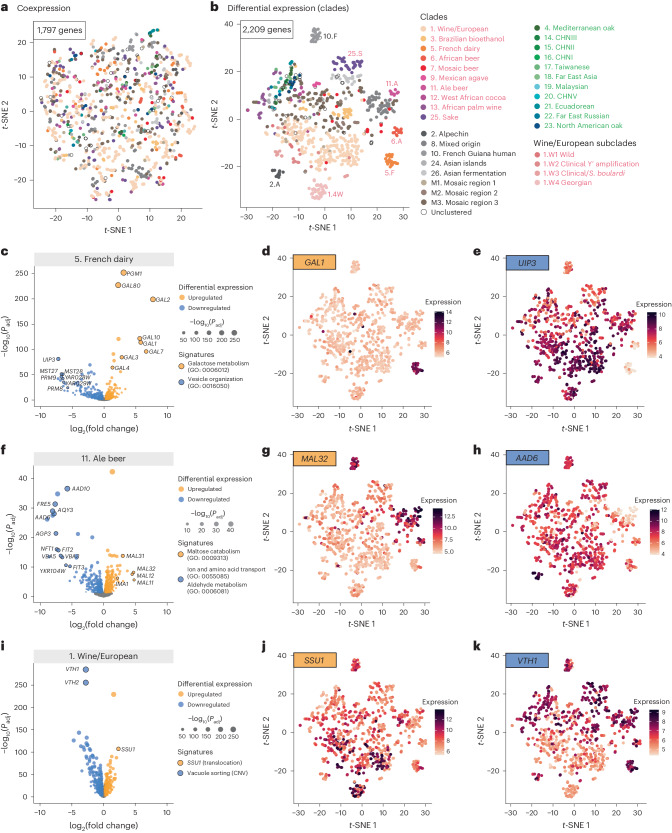
Subpopulation-specific differential gene expression. **a**,**b**, Isolate-level clustering using *t*-SNE based on the expression of 1,797 coexpressed genes (**a**) and 2,209 DEGs (**b**). Isolates belonging to each annotated subpopulation are color-coded. **c**–**k**, Examples of subpopulation-specific domestication signatures. Three examples are shown. **c**, 5. French dairy. **d**, 5. French dairy *GAL1*. **e**, 5. French dairy *UIP3*. **f**, 11. Ale beer. **g**, 11. Ale beer *MAL32*. **h**, 11. Ale beer *AAD6*. **i**, 1. Wine/European. **j**, 1. Wine/European *SSU1*. **k**, 1. Wine/European *VTH1.* In **c**,**f**,**i**, the volcano plots show upregulated (orange) and downregulated (blue) genes and biological processes, with the *x* axis showing the log_2_ fold change when the subpopulation was compared with the rest of the isolates, and the *y* axis showing the Benjamini–Hochberg-adjusted −log_10_*P*_adj_. The dot sizes were scaled according to the FDR. The variance-stabilized expression levels for specific upregulated (**d**,**g**,**j**) and downregulated (**e**,**h**,**k**) genes were overlapped over the *t*-SNE plot, with expression levels scaled as different color intensities. The colored scales are shown on the side of the plots.

While the coexpression network reflected globally coordinated cellular processes at the population level, the differential expression set revealed variability in subpopulation-specific gene expression. To further characterize this aspect, we looked at the clustering of the individual isolates based on expression across the coexpression set (1,797 genes) and the differential expression set (2,209 genes), using *t*-distributed stochastic neighbor embedding (*t*-SNE) (Fig. 4a,b). As expected, no structure related to the subpopulations was defined using *t*-SNE on coexpressed genes (Fig. 4a). By contrast, a clear delineation of subpopulations, including multiple domesticated clades, was observed using *t*-SNE on DEGs (Fig. 4b). Specifically, domesticated clades such as Wine/European (1.W), French dairy (5.F), African beer (6.A), Ale beer (11.A) and Sake (25.S), all showed a clear and distinctive delineation, suggesting independent transcriptional signatures that are unique to each domestication process (Fig. 4b). The mosaic subpopulations (M1–M3 and unclustered) and the Brazilian bioethanol clade (3.B) showed a more scattered pattern, which was consistent with their admixed genome structure. Interestingly, all the wild subpopulations, despite their high genetic divergence, showed little transcriptomic differentiation and were all closely clustered together (Fig. 4b). The West African cocoa (12.W) and the African palm wine (13.A) clades, although involved in human-related fermentation processes, are derived directly from wild lineages and clustered more closely to the wild subpopulations (Fig. 4c). The *t*-SNE clustering was further corroborated by the topologies of the neighbor-joining trees (Supplementary Fig. 7a–c) based on Euclidean distances among isolates using either the coexpression (Supplementary Fig. 7b) or differential expression gene sets (Supplementary Fig. 7c). These observations showed that the wild populations did not display differentiated expression patterns despite their high genetic divergence, suggesting that the differential expression landscape was mainly driven by multiple distinctive domestication processes in our experimental settings.

To further characterize these transcriptional signatures, we performed GSEA based on the ranked log_2_ fold change of the DEGs in each subpopulation (Supplementary Fig. 8 and Supplementary Table 8). Significant enrichment for several biological processes was found across different clades, most of which were adaptive in specific domestication processes (Supplementary Fig. 6c). For example, genes in the *GAL* pathway, involved in the metabolism of galactose, were significantly upregulated in the French dairy clade (5.F) (Fig. 4c). In this subpopulation, the *GAL* pathway went from a tightly regulated glucose-repressed and galactose-induced system to constitutive expression, even in the presence of glucose. Such switching was previously found in several lineages involved in spontaneous milk fermentation and was linked to adaptation to a lactose-rich medium^24,25^. In addition to the *GAL* genes (Fig. 4c,d), the French dairy clade also showed downregulation of multiple putative integral membrane proteins in the DUP240 family (for example, MST27, MST28 and UIP3) that are involved in COPI-related and COPII-related vesicle organization^26^ (Fig. 4c–e). Such changes in cell secretion could also be adaptive to certain cheese-making processes^27^. In several types of alcohol fermentation, adaptive transcriptional signatures were also prevalent (Fig. 4f–h, Supplementary Fig. 6c and Supplementary Table 8). For example, the *MAL* genes involved in maltose catabolism are upregulated in the Ale beer clade (11.A), a signature associated with the malt fermentation environment in beer making^28^ (Fig. 4f,g). At the same time, the expression of multiple aldehyde dehydrogenase genes (such as *AAD6* and *AAD10*) was downregulated in the Ale beer cluster (Fig. 4f–h). Similarly, downregulation of another aldehyde dehydrogenase gene, *ADH7*, was seen in the Sake (25.S) cluster, along with a pathway-level upregulation of genes involved in thiamine metabolism (Supplementary Fig. 8 and Supplementary Table 8). Both these downregulated and upregulated signatures ensure high ethanol yield during sake production^29^. Another well-known adaptive trait in certain wine isolates involved several translocations that led to the overexpression of *SSU1*, a sulfite pump that confers resistance to sulfur dioxide, a commonly used compound in wine making^30,31^. Overexpression of *SSU1* was indeed seen in the Wine/European clade (1.W) in our dataset (Fig. 4i–k).

Differential expression analyses highlighted transcriptional signatures specific to different subpopulations. Interestingly, wild subpopulations were less differentiated in terms of transcriptional diversity, despite the high level of genetic divergence among these clades under standard laboratory conditions. In contrast, domesticated subpopulations exhibited clear signatures that corresponded to distinct adaptive processes, notably in several metabolic pathways uniquely selected in different domestication events.

### Introgression, horizontal gene transfer and expression variation

The recently established *S. cerevisiae* pangenome revealed many horizontally transmitted evolutionary events, such as introgressions and horizontal gene transfers (HGTs), as part of the accessory genome^20^. This part of the pangenome was also more specific to certain subpopulations. For example, the presence of *Saccharomyces paradoxus* introgression events was a main characteristic of the Alpechin (2.A), Mexican agave (9.M) and French Guiana (10.F) clades^20^. By contrast, the Wine/European (1.W) clade featured many HGT events from the *Torulaspora* and *Zygosaccharomyces* species^20^.

**Fig. 5:**
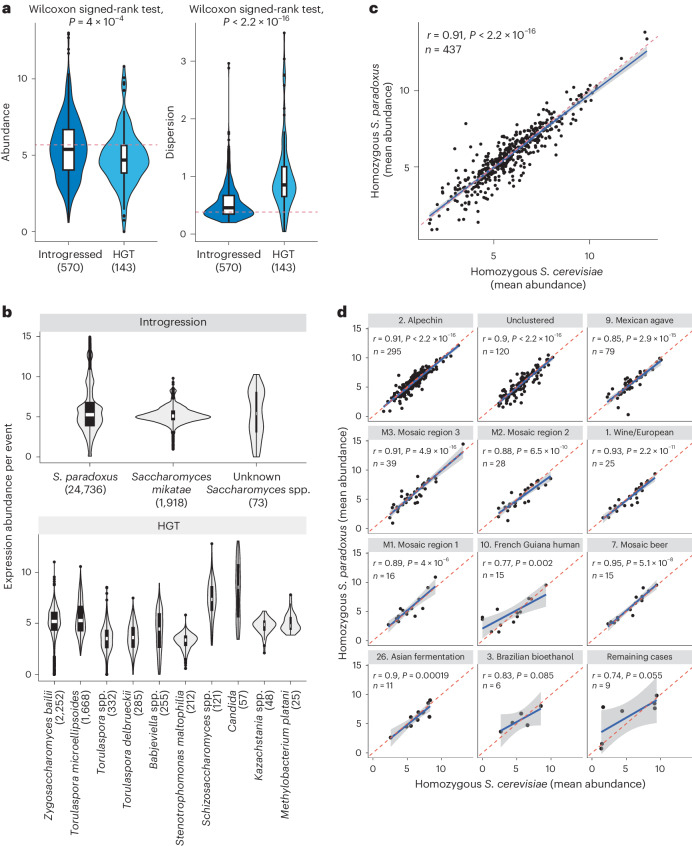
Global expression patterns for accessory gene subcategories. **a**, Mean expression abundance and dispersion for introgressed (*n* = 570) and HGT genes (*n* = 143). *P* values were determined using a two-sided Wilcoxon rank-sum test. The red dashed line indicates the median value observed in core genes. **b**, Expression abundance per event for the introgression and HGT subcategories according to donor species. Expression abundance was calculated as log_2_(TPM + 1). The middle bar of all box plots corresponds to the median; the upper and lower bounds correspond to the third and first quartiles. The whiskers correspond to the upper and lower bounds 1.5 times the IQR. **c**, Expression correlation between 437 homozygous introgressed genes from *S. paradoxus* and the corresponding *S. cerevisiae* version. The axes show the mean abundance as log_2_(TPM + 1) either in strains with homozygous *S. paradoxus* introgressions (*y* axis) or in strains with the homozygous *S. cerevisiae* version for the same genes. The dashed line indicates the 1:1 ratio. The Pearson’s correlation coefficient and *P* value are shown. **d**, Expression correlation for *S. paradoxus* introgression versus the *S. cerevisiae* counterparts across subpopulations. The panels are arranged in descending order based on the number of homozygous introgressed genes in each subpopulation. The last panel regroups seven subpopulations with one or two genes only. The dashed line indicates the 1:1 ratio. The Pearson’s correlation coefficient and *P* value are shown.

While expression of the accessory genome was globally characterized by low abundance and high dispersion, further differences were observed across different accessory gene subcategories. Specifically, introgressed genes displayed higher expression abundance and lower dispersion compared with genes originated from HGTs (Fig. 2d and 5a). These differences can potentially be attributed to the origin species for these genes (Fig. 5b). In fact, regardless of the origin of the introgressed genes, the level of gene expression was globally similar (Fig. 5b). In contrast, there was large disparity in gene expression levels according to the origin species for the HGT events, leading to higher dispersion (Fig. 5b).

The vast majority of introgressed ORFs examined in this study comes from *S. paradoxus*, a *S. cerevisiae* sister species. In most cases, these genes substitute their *S. cerevisiae* ortholog, either partially, resulting in a heterozygous state (one allele from each species), or completely, resulting in a homozygous state. We first examined the expression of 437 genes homozygous for either the allele of *S. cerevisiae* or that of *S. paradoxus* in different strains and found that their expressions are well correlated (Fig. 5c,d and Supplementary Fig. 9a). We then examined the effects of such introgressions in heterozygous states using allele-specific expression (ASE) analyses (Methods, datafile 3). Again, we found no significant differences in expression between the *S. cerevisiae* and the *S. paradoxus* alleles (Supplementary Fig. 9c). Overall, the introgressed alleles of *S. paradoxus were* expressed at a level similar to those of *S. cerevisiae*, suggesting that they were well integrated into the transcriptional network.

### Genetic basis underlying the pan-transcriptome variation

**Fig. 6:**
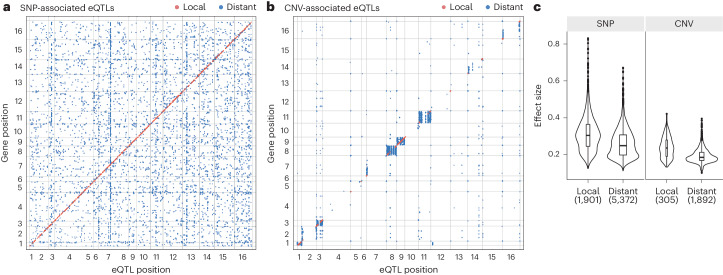
GWAS-identified SNPs and CNVs with their associated eQTLs. **a**, Locations of local and distant SNP-associated eQTLs along the genome. SNP variants associated with an expression trait are on the *x* axis; genes are shown on the *y* axis. Local eQTLs are shown in red and distant eQTLs in blue. **b**, Locations of local and distant CNV-associated eQTLs. Local eQTLs correspond to associations between the CNV and the expression trait of the same gene and are shown in red. Distant eQTLs are shown in solid blue. **c**, Comparison of the absolute effect sizes for local and distant eQTLs for SNP-associated and CNV-associated eQTLs. *P* values were determined using a two-sided Wilcoxon rank-sum test. Sample sizes are shown on the plots. The center of the box plots corresponds to the median; the upper and lower bounds correspond to the third and first quartiles. The whiskers extend to the upper and lower bounds 1.5 times the IQR (Supplementary Fig. 7).

To further understand the relationship between pangenome variation and the transcriptional landscape, we performed GWAS by considering both the SNPs and CNVs that were previously characterized in the population^20^. Across the 969 isolates, 84,682 SNPs and 1,100 CNVs were included, with a minor allele frequency (MAF) higher than 5%. A total of 9,470 significant eQTLs were detected (Methods). In total, significant eQTLs were associated with the expression variation of 3,471 genes. Among the detected eQTLs, 7,273 were associated with SNPs and 2,197 were associated with CNVs, corresponding to 4,393 and 497 unique loci, respectively (Fig. 6a,b, datafile 4).

Regarding SNP-associated eQTLs, 1,901 were local eQTLs, with sites located in upstream of the transcription start site or within the ORF of the target gene displaying the largest effect sizes (Supplementary Fig. 10a,b). The remaining 5,372 SNP-associated eQTLs were distant and *trans*-acting. Overall, local SNP-associated eQTLs were less frequent, representing ~26% of the total set of eQTLs detected, which is consistent with previous findings based on linkage mapping across a large segregant panel in a yeast biparental cross^6^. The *trans*-eQTLs detected were uniformly distributed across the genome, with ~41% of eQTLs (2,206 of 5,372) impacting only one trait (Fig. 6a). The most significant eQTL hotspot was mapped to the *CTT1* gene, encoding for a cytosolic catalase T, which is associated with 251 expression traits and is an eQTL hotspot under stress conditions^32^ (Supplementary Fig. 10c). Contrasting previous observations in a yeast cross^6^ and recent findings in a *Caenorhabditis elegans* population^5^, the set of *trans*-eQTLs detected in our large dataset were not biased toward a few hotspots with extreme pleiotropic effects (Supplementary Fig. 10c).

Unlike SNPs, the effect of CNVs on variation in gene expression has never been systematically explored at the species scale. Compared with SNPs, CNVs are not randomly distributed along the genome and are located toward the subtelomeric regions, except for chromosomes 1 and 9 because of the presence of aneuploidies that passed the 5% MAF filter (Fig. 6b). In addition, chromosomes 3, 8 and 11 are also impacted by aneuploidies at a lower frequency (~3%), resulting in a larger number of CNV-associated eQTLs in those regions (Supplementary Fig. 10d). These aneuploid chromosomes artificially inflate the *trans* hotspots for CNV-associated eQTLs (Supplementary Fig. 10d). We only considered CNV-associated eQTLs to be local when the CNV was directly associated with the same gene expression trait, and distant CNV-associated eQTLs corresponding to single linkage groups. This resulted in a total of 305 local CNV-associated eQTLs versus 1,892 distant CNV-associated eQTLs. On average, each CNV-associated eQTL impacts about eight expression traits.

Consistent with previous observations, local SNP-associated eQTLs displayed larger effect sizes compared with distant ones, with a 1.3-fold higher absolute effect size and 2.4-fold higher variance explained on average (Wilcoxon signed-rank test, *P* < 2.2 × 10^−16^; Fig. 6c and Supplementary Fig. 10e). While the same trend held true for CNV-associated eQTLs for absolute effect size (1.2-fold higher absolute effect size, local versus distant, Wilcoxon signed-rank test, *P* < 2.2 × 10^−16^; Fig. 6c), the variance explained by local or distant CNV-associated eQTLs was low and not significantly different because of an overall lower MAF of CNVs compared with SNPs (Supplementary Fig. 10e). Overall, CNV-associated eQTLs displayed smaller effect sizes compared with SNP-associated eQTLs across the board. This first direct comparison of eQTL effect sizes indicated that SNPs had a significantly larger impact than CNVs for variation in gene expression at the population level.

From a functional perspective, *trans*-eQTLs uncovered coherent associations that link causal SNPs and gene expression traits within the same biological process (Supplementary Fig. 10f). The top five *trans*-eQTL hotspots collectively impacted the expression of 356 genes, of which 276 belonged to the ribosome biogenesis module in the coexpression network (Supplementary Fig. 10f). The causal SNPs mapped to *CTT1* (251 eQTLs, chromosome 7, position 655851), *SRD1* (84 eQTLs, chromosome 3, position 148921), *DHR2* (82 eQTLs, chromosome 11, position 290740), *RAD52* (71 eQTLs, chromosome 13, position 213896) and *RPS17A* (62 eQTLs, chromosome 13, position 225572) (Fig. 6b), of which *SRD1* and *DHR2* were involved in rRNA processing and synthesis, and *RPS17A* (associated with a ribosomal protein), all of which were directly related to ribosomal biogenesis. Furthermore, *trans-*eQTL hotspots affected disproportionally more genes in the coexpression network. Among the eQTL hotspots associated with more than 20 traits, the expression of 648 genes was affected, of which 404 belonged to the coexpression network versus 105 that belonged to DEGs (Supplementary Table 9).

By integrating the eQTL results with the global transcriptome structure, we uncovered distinctive patterns regarding the genetic basis underlying coexpression and DEGs (Supplementary Fig. 11a–c). Overall, DEGs are significantly more likely to be controlled by any eQTL compared with the total set (odds ratio (OR) = 1.1, two-sided Fisher’s test, *P* = 0.01; Supplementary Fig. 11a), while coexpression genes were slightly depleted (OR = 0.92, Fisher’s test, *P* = 0.08). However, the types of eQTL involved showed more drastic differences, with a 0.38-fold depletion of local eQTLs (two-sided Fisher’s test, *P* < 2.2 × 10^−1^) for coexpression genes and a 1.42-fold enrichment of local eQTLs in DEGs (two-sided Fisher’s test, *P* = 1.921 × 10^−8^; Supplementary Fig. 11b). CNV-associated eQTLs were also significantly depleted in coexpression genes (OR = 0.62, two-sided Fisher’s test, *P* = 4.553 × 10^−7^) (Supplementary Fig. 11c).

**Fig. 7:**
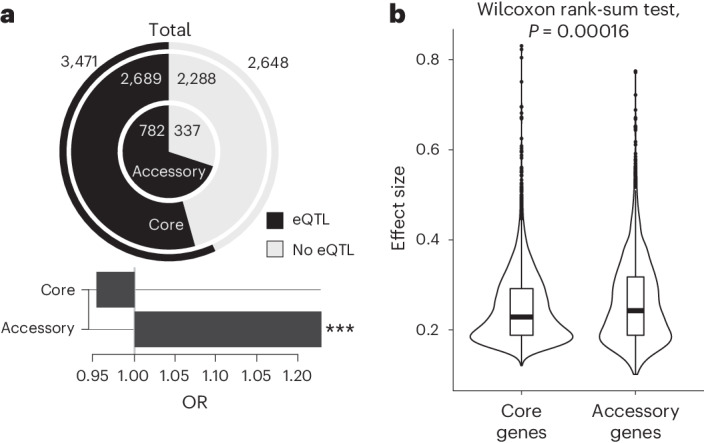
Accessory genes show proportionally more eQTLs with higher effect sizes. **a**, Number and proportion of genes impacted by at least one eQTL for accessory genes (inner ring), core genes (middle ring) and the combined set (outer ring). Fold enrichment for core and accessory genes on the proportion of genes impacted compared with the combined set are presented as bars (OR). Significant enrichment based on a two-sided Fisher’s test is indicated by the three asterisks (OR = 1.33, two-sided Fisher’s test, *P* = 0.0002676). **b**, Comparison of the absolute effect size for eQTLs associated with accessory or core gene expression traits. *P* values were determined using a two-sided Wilcoxon rank-sum test. Sample sizes are shown in the plots. The center of the box plots corresponds to the median; the upper and lower bounds correspond to the third and first quartiles. The whiskers correspond to the upper and lower bounds 1.5× the IQR. *n* = 3,378 for associations with accessory genes and *n* = 6,092 for associations with core genes (see also Supplementary Fig. 11).

From the perspective of the pangenome, accessory genes were significantly more likely to be controlled by at least one eQTL compared with core genes (Fig. 7a). Accessory genes were also significantly more likely to be controlled by local eQTLs (OR = 1.33, two-sided Fisher’s test, *P* = 0.0002676) (Supplementary Fig. 11d,e). Most remarkably, the effect size for eQTLs associated with accessory genes was globally higher compared with core genes (Fig. 7b); the same trend was true for the fraction of variance explained (Supplementary Fig. 11f). These differences were not biased toward accessory genes with a low occurrence in the population (Supplementary Fig. 12). Overall, these observations clearly showed that the accessory genome is a key component of the regulation of variation in gene expression at the population scale.

## Discussion

The species-wide pan-transcriptomic analysis presented in this study has led to a precise characterization of the functional organization and genetic basis underlying the transcriptional landscape at a scale that, to our knowledge, has yet to be achieved in any other species. Our results revealed the accessory genome as a key driver of transcriptional diversity, contributing proportionally more to variation in heritable expression than the core genome.

The natural population of *S. cerevisiae* is highly diverse and has a clear population structure, with defined subpopulations assigned to specific domesticated and wild lineages^24,33,34^. Such population structure is commonly observed in several species, including humans^18^; however, the impact of population structure on the transcriptional landscape is largely unclear. We characterized gene expression patterns both at the whole-population level using coexpression analysis, and at the subpopulation level using differential expression analysis. Our results show that the global transcriptional landscape is consistent with a two-tier architecture, characterized by a tightly interconnected central network (that is, coexpression) and an auxiliary structure related to differential gene expression patterns (that is, differential expression). These two architectural levels are not equally impacted by the structure of the population. On the one hand, the coexpression network captures the main biological functions, reflects the topological organization of the cell and is globally conserved across subpopulations. On the other hand, differential expression reveals subpopulation-specific, functionally coherent upregulation and downregulation associated with distinct domestication signatures. The robustness of the coexpression network to genetic variation in the population suggests a buffering effect at the transcription level to maintain the expression level of genes involved in core cellular functions^35,36^.

From the pangenomic perspective, the accessory genome, including ancestrally segregating genes in the *S. cerevisiae* species and horizontally acquired genes from close (introgressed) or distant (HGT) relatives, all exhibited higher expression dynamics compared with the core genome. The core and accessory gene features roughly echo the two-tier transcriptomic landscape, with accessory genes more likely to be involved in the auxiliary network than in the central one. In addition, expression patterns suggest that accessory genes, despite being variable across the population, can also be important to certain adaptive processes and represent an integral part of the functional genome.

The large population size and the fully cataloged genetic variants allowed us to systematically explore the genetic basis underlying transcriptional variation at the species level. By performing GWAS with both SNPs and CNVs, we uncovered local and distant eQTLs for over 56% of the expression traits, with SNP-associated eQTLs explaining a significantly higher fraction of variance compared with CNV-associated eQTLs. Overall, local eQTLs explained a higher fraction of variance, which is consistent with previous observations^5,6^. However, distant eQTLs were mostly randomly distributed along the genome and were not biased toward a few extreme hotspots, unlike a previous observation in a single cross^6^. As these hotspots were possibly related to large-effect rare variants with extreme pleiotropy, it is not surprising that such a pattern is not conserved in a large natural population where genomic constitution is much more complex. Finally, accessory genes were significantly more likely to be associated with eQTLs than core genes. Moreover, eQTLs associated with accessory genes also explained a higher fraction of the variance in expression.

Our analyses at all levels collectively show the surprisingly high impact of accessory genes on the transcriptional landscape within a species. Therefore, a better view of the pangenome, and more specifically, the accessory genome is the next step needed to efficiently target molecular traits that are more susceptible to genetic variation between individuals, including traits with clinical relevance. While the accessory genome probably explains some of the missing heritability, understanding genetic effects on cellular phenotypes is far from complete. Dissecting the genetic regulation of an additional set of molecular phenotypes or intermediates, such as proteomes, will most probably yield additional insights.

## Methods

### Description of the isolates and sample preparation

A collection of 1,032 isolates was compiled from the 1,011 strains in the collection^20,22^, along with the laboratory reference strain S288C (FY4-6). We measured growth in all strains using 96-well liquid growth in standard synthetic complete medium with 2% glucose as the carbon source. Growth rates were then extrapolated based on continuous optical density (OD) measurements for 48 h at 30 °C in a microplate reader (Infinite F200 Pro, Tecan). The strains were reorganized and grouped in 96-well plates according to their growth rates and then grown in 1 ml of liquid standard synthetic complete medium using deep well blocks until the mid-log phase was reached (OD ~0.3). For each sample, 750 µl of the culture was collected and then transferred to a sterile 0.45-µM, 96-well filter plates (cat. no. 40008, Norgen) on a multi-well plate vacuum manifold (cat. no. 16003-836, VWR). We applied a vacuum to remove all the standard synthetic complete medium, sealed the plate with aluminum foil seals, and flash-froze the entire plate in liquid nitrogen to store the plate at −80 °C before mRNA extraction. A final set of 969 isolates was included in our dataset after controlling for the final OD reading at the culture collection step and the quality of the RNA-seq data. A detailed description of the isolates can be found in Supplementary Table 1.

### RNA extraction, library preparation and sequencing

For each filter plate, mRNA was extracted using the Dynabeads mRNA DIRT Purification Kit (cat. no. 61012, Thermo Fisher Scientific) based on an optimized protocol for high-throughput RNA-seq^6^. Cells were lysed using glass beads and lysis buffer, then incubated for 2 min at 65 °C. After RNA precipitation, two rounds of cleaning were performed using magnetic beads coupled to oligo (dT)_25_ residues which can hybridize to the poly(A) tail of the mRNA. A final volume of 10 µl purified mRNA was obtained to prepare the sequencing library.

Sequencing libraries were prepared with the NEBNext Ultra II Directional RNA Library Prep Kit with Sample Purification Beads (cat. no. E7765L, New England Biolabs) in 96-well plates. We used 5 µl purified mRNA for library preparation, corresponding to ~10 ng of RNA molecules per sample. We generated complementary DNA (cDNA) libraries using reverse transcription. The resulting cDNA libraries were then purified using NEBNext sample purification magnetic beads and eluted in 50 µl 0.1× Tris-EDTA buffer. Dual index duplex adapters were added to the cDNA using ligation. In total, 96 combinations of TS HT dual index, duplex-mixed adapters from Integrated DNA Technologies were used and each prepped DNA was assembled to a unique barcode combination. Adapter-ligated cDNA was purified using NEBNext sample purification magnetic beads and eluted in 15 µl 0.1× Tris-EDTA buffer. A final PCR enrichment of the barcoded DNA was performed in a 9-cycle amplification using Illumina P5 and P7 universal primers (Integrated DNA Technologies) (P5: 5′-AATGATACGGCGACCACCGA-3′; P7: 5′-CAAGCAGAAGACGGCATACGA-3′). Then, 21 µl of the final barcoded DNA were purified and eluted in 0.1× Tris-EDTA buffer.

For each sample, the final barcoded DNA was quantified using the Qubit dsDNA HS Assay Kit (Invitrogen) in a 96-well plate with a microplate reader (Infinite F200 Pro), with the excitation laser set at 485 nm and the emission laser at 528 nm. All samples from the 96-well plate with a concentration higher than 1 ng µl^−1^ were grouped; 20 ng of cDNA were collected and pooled from each sample. The DNA integrity of the pool was controlled on 1% agarose gel and quantified on NanoDrop and Qubit using the Qubit dsDNA HS Assay Kit.

The final pool of DNA was sequenced on the NextSeq 550 high output at the European Molecular Biology Laboratory Genomics Core Facilities; 1,046 samples were sequenced, including duplicates for 29 of the isolates (datafile 2). On average, 6.45 million of 75-bp single-end reads were obtained for each sample after demultiplexing (Supplementary Table 1).

### Read cleaning and data processing

Raw reads were cleaned with cutadapt (v.1.8.1)^37^ to remove adapters and low-quality reads, which were trimmed based on a Phred score threshold of 30 and discarded if less than 40-nt long after this trimming step.

For each of the 1,046 samples, clean reads were mapped to the *S. cerevisiae* reference sequence using TopHat (v.2.0.13)^38^. The resulting BAM files were sorted and indexed using SAMtools (v.1.9)^39^. Duplicated reads were marked using Picard (v.2.18.14) in the Genome Analysis Toolkit (GATK) v.4.1.0.0 (ref. ^40^). HaplotypeCaller was used to call variants in each individual sample. The variant calling files (VCFs) were merged and rare SNPs, defined as having an MAF less than 5% were extracted and intersected with SNP data from ref. ^20^ using bcftools isec.

The 1,046 samples were ranked based on the number of shared rare SNPs with each relevant strain described in the SNP matrix. This allowed automatic validation of 940 unique isolates for which the expected strain was among the top three ranking strains. The remaining samples were investigated manually: 24 samples that were part of a large cluster of closely related strains could be validated as the expected strain; 19 samples could be unambiguously reassigned to the top one ranking strain; and 14 of 1,046 samples could not be validated or reassigned and were discarded from the remaining analyses. After this step, a final set of 987 unique isolates was retained.

### Gene expression quantification

For each validated sample, reads were mapped to the *S. cerevisiae* reference sequence for which the SNPs of the corresponding strains were inferred (as described in ref. ^20^) plus the accessory genes that were not classified as ancestral or orthologs of *S. paradoxus* in ref. ^20^ (*n* = 395). The mapping was achieved using STAR (v.2.5.2b)^41^ with the following parameters: --outReadsUnmapped Fastx--outSAMtype BAM SortedByCoordinate--outFilterType BySJout--outFilterMultimapNmax 20--outFilterMismatchNmax 4--alignIntronMin 20--alignIntronMax 2000--alignSJoverhangMin 8--alignSJDBoverhangMin 1. Isolates with more than 1 million mapped reads were kept for analysis, resulting in a final set of 969 strains (Supplementary Table 1).

Mapped read counts were then obtained using the featureCounts function from the Subread (v.2.0.2) package^42^, with the genes described in the *S. cerevisiae* reference annotation (*n* = 6,285) and accessory genes (*n* = 395) as features. The following options were used to count multi-mapped reads as a fraction of the sites they mapped to: -M --fraction.

Finally, TPM were calculated as a measure of transcription abundance for each of those features and a log_2_(TPM + 1) transformation was applied. From the set of 6,285 reference genes, 196 were filtered out because log_2_(TPM + 1) was lower than 1 in 50% of isolates. The read counts for 39 accessory features with a known homolog in *S. cerevisiae* according to the pangenome annotations were merged with the corresponding homolog. Thus, the final set consists of 6,445 ORFs that were used for the downstream analyses (Supplementary Table 2).

### Neighbor-joining tree

The VCFs of the 969 final strains were combined using GenotypeGVCFs in GATK (v.4.1.0.0). Biallelic segregating sites were used to construct a neighbor-joining tree with the R packages ape^43^ and SNPrelate^44^. Briefly, the gvcf matrix was converted into a GDS file for individual dissimilarities to be estimated for each pair of individuals using the snpgdsDiss function. The BIONJ algorithm was then run on the obtained distance matrix.

### Calculating mean expression abundance and dispersion

In total, 944 of the 969 isolates in the final dataset were present in the 1,011 isolates characterized previously^20^. For these isolates, pangenome annotations in terms of the presence and absence of a given gene in each isolate are available. We used this set of isolates and their expression levels to calculate the mean expression abundance and dispersion. Abundance corresponds to the mean expression levels of all isolates where the gene is annotated as being present. Dispersion is calculated as the MAD using the following formula:

$$\frac{1}{n}\mathop{\sum }\limits_{i=1}^{n}{\rm{|}}{x}_{i}-\bar{x}{\rm{|}}$$

Where $n$ is the number of strains that carries the gene, ${x}_{i}$ is the expression level in log_2_(TPM + 1) for the *i*th isolate and $\bar{x}$ is the mean log_2_(TPM + 1) for all samples for a given gene. Genes present in only one isolate were excluded. Genes not expressed in any isolate were also excluded. In total, 6,138 genes passed the filters and were included in the analysis, including 1,291 accessory and 4,847 core genes. All annotations can be found in datafile 1.

### Variance-stabilizing transformation

We performed variance-stabilizing transformation using the raw counts for each gene across the 969 isolates. We excluded genes that were not expressed in over half of the samples, which eliminated most accessory genes originated from HGT. The remaining 6,119 genes were normalized using the vst() function in the R package DEseq2 (ref. ^45^). The variance-stabilized expression values were subsequently used for the coexpression and differential expression analyses.

### Gene coexpression analysis and module detection

We calculated the Pearson’s correlation between all pairwise combinations in the expression of the 6,119 variance-stabilized genes. We generated an adjacency matrix by removing any gene pairs with an absolute correlation coefficient of less than 0.67, then created an undirected network graph using the igraph package in R. We calculated the connectivity for each node and recursively removed nodes connected by fewer than five edges. This resulted in a final graph containing 1,797 nodes and 181,954 edges. Graphic representation of the network was calculated using the Fruchterman–Reingold layout in the sna package in R^46^.

To detect the coexpression modules, variance-stabilized expression of the 1,797 node genes was used to generate a topological overlap matrix (TOM) using the R package WGCNA^47^. We performed a scale independence test and determined the soft-threshold *β* value, also known as the power value. At a *β* of 5, the scale-free topology model fit was stabilized at an *R*^2^ = 0.9. Therefore, the TOM was calculated based on the signed adjacency matrix with a power of five, using the TOMsimilarity() function in WGCNA. The dissimilarity matrix was then calculated as 1 − TOM.

A distance matrix based on the TOM dissimilarity was calculated using the as.dist() function. Clustering was performed using hclust() in the fastcluster package, with the ‘average’ method^48^. We used the cutreeDynamic() function in the dynamicTreeCut package^49^ to determine topologically independent clusters, with the options cutHeight = 0.95 and minClusterSize = 5. These clusters were treated as pre-modules for which ME expression was determined using WGCNA^47^. This pre-ME expression was clustered based on the dissimilarity of the pairwise correlation matrix, again using hclust() with method = ‘average’. Based on this, eigengenes with a dissimilarity lower than 0.2 were merged, forming a final set of 16 modules (Supplementary Table 4). GO term enrichment analyses were performed using annotations for both biological process and cellular compartment standards^50^, using the mod_ora() function in the CEMiTool package^51^. Detailed enrichment results are shown in Supplementary Table 5.

For the final 16 modules, eigengene expression was calculated using the function moduleEigengenes() in WGCNA. PCA based on eigengene expression was performed using the prcomp() function in the stats package.

### Subpopulation-specific differential expression analyses

The variance-stabilized expression dataset consisting of 6,116 genes was used to perform subpopulation-specific differential expression analyses using DEseq2 (ref. ^45^). Each subpopulation was compared with the remaining isolates using the expression matrix and annotated isolate information. Around 10% of the isolates in our dataset were haploids with defined mating types. To eliminate the effect of the mating type-specific expression signature, we incorporated the mating type information in the design model as a covariate. Significant differential expression was determined using a Benjamini–Hochberg-adjusted *P* value of less than 5%, corresponding to a 5% FDR.

Because of the imbalanced subpopulation sizes, the FDR cutoff alone was biased toward detecting more differential expression, with small effect sizes in larger subpopulations. To remove this bias, we repeated the analyses by setting a cutoff on the absolute log_2_ fold change ranging between 0 and 1, with an increment of 0.05. We counted the number of significant hits based on each cutoff and evaluated its relationship with the size of the subpopulation. We found that the dependency between the number of significant hits and subpopulation size was removed around a cutoff of an absolute log_2_ fold change of 0.2–0.3. Therefore, we chose an absolute log_2_ fold change greater than 0.3 and an adjusted *P* value of less than 5% as the final criteria for significant hits. All significant differential expression hits are presented in Supplementary Table 6. Hits that overlapped with coexpression genes were mainly associated with the Ecuadorean (21.E) and French Guiana human (10.F) subpopulations; they were not included in the differential expression gene set for the subsequent analyses.

### GSEA

GSEA was performed for the gene-level abundance and dispersion analyses (Supplementary Fig. 1), coexpression module overrepresentation (Supplementary Fig. 3b) and for the differential expression sets (Supplementary Fig. 3c).

GSEA for expression abundance and dispersion was performed using the fgsea R package^52^. Annotation of the GO Slim terms was obtained from the *Saccharomyces* Genome Database. To reduce redundancy, we used the rrvgo R package and calculated the similarities between each annotated term in a pairwise manner using the ‘Resnik’ method, and removed terms that were at least 70% overlapping with another term. We then performed GSEA using the fgsea() function, the pathways corresponding to the reduced GO Slim terms and a size limit on the terms between 50 and 600; 100,000 permutations were performed. The results are shown in Supplementary Table 3.

We performed a GSEA-based module overrespresentation test using the mod_gsea() function in CEMiTool^51^ to test for subpopulation-related differential coexpression. Subpopulations with fewer than ten isolates were removed to ensure statistical power. The results are shown in Supplementary Table 7.

Finally, GSEA was performed to identify GO term enrichment for subpopulation-specific differential expression. For each subpopulation, significant hits were ranked according to the log_2_ fold change and then tested for enrichment in standard GO terms for annotated biological processes, with term size limits between five and 500. For each test, 10,000 permutations were performed. The results are shown in Supplementary Table 8.

### ASE

We selected all isolates previously described as diploid, euploid and heterozygous^20^ to perform ASE analysis on this population (*n* = 289). We quantified the biallelic expression of each of these isolates using the GATK tool ASEReadCounter^53^ by providing for each isolate a BAM file resulting from an alignment of RNA-seq reads on the reference genome and a VCF file containing all heterozygous positions of the corresponding isolate. Heterozygous sites displaying a risk of allelic mapping bias were detected using their 75-bp mappability from GenMap^54^. We used the allele count to calculate an alternative allele ratio (AAR):

$$\frac{{\mathrm{Alternative}}\; {\mathrm{allele}}\; {\mathrm{counts}}}{{\mathrm{Reference}}\; {\mathrm{allele}}\; {\mathrm{counts}}+{\mathrm{alternative}}\; {\mathrm{allele}}\; {\mathrm{counts}}}$$

We finally excluded sites that did not have their heterozygosity supported by their AAR (AAR = 0 or 1).

We detected imbalance in allele expression using a simple binomial test corrected using the FDR. To further compensate residual mapping bias in our results, we set the probability value of the binomial test to the mean of the AAR in all 289 isolates instead of 0.5. Moreover, we performed the previous test on sites that were covered more than 29 times to ensure enough statistical power for the binomial test. Finally, we limited our explorations of ASE to the heterozygous sites located in coding sequences. A list of 214,551 heterozygous sites distributed in 3,570 unique genes was analyzed across all 289 isolates (median = 464 sites per isolate).

For heterozygous introgression cases with *S. cerevisiae* and *S. paradoxus* alleles, the unfiltered VCF files from ref. ^20^ were corrected for coverage and mapping bias, resulting in 3,338 sites related to heterozygous introgressed genes. A significant difference was found regarding AAR between these 3,338 introgressed sites and non-introgressed sites toward low values for introgression. However, among those sites, some were displaying aberrant genetic allele balance (AB) (AB tag in the VCF file) because of soft filtration. Thus, we iteratively performed several filtration steps of genetic AB. Briefly, at each step, the filtration value was set to exclude extreme genetic AB: for example, with a filtration value of 0.1, sites with a genetic AB higher than 0.9 or lower than 0.1 were discarded; for 0.2, the threshold was 0.8 and 0.2. Ultimately, this led to selecting sites with genetic AB narrowed to 0.5 but also resulted in an important decrease in the number of sites. In addition, at each filtration step, we compared the AAR between heterozygous introgressed sites and non-introgressed sites and found that the AAR difference between introgressed sites and other sites decreased as the filtration value increased. Because extreme genetic AB could be related to a difference in allele copy number, and because our goal was to compare the AB in genes with similar genetic organization in their alleles, we selected sites with a genetic AB between 0.33 and 0.66. This resulted in 356 sites distributed in 43 heterozygous introgressed genes. All data can be found in datafile 3.

### GWAS

GWAS based on mixed-model association analysis were performed as described in ref. ^20^ using FaST-LMM^55^. To detect SNP-associated eQTLs, we removed the subtelomeric regions corresponding to 20 kb each side of the chromosomes from the SNP matrix; 84,682 SNP sites and 1,100 CNVs between 969 strains with an MAF higher than 5% were tested. For CNV-associated eQTLs, as most variants were located in the subtelomeric regions, all expression traits were included. The variation in expression (in TPM) of 6,119 genes was tested. The SNP matrix was used for kinship for both the SNP and CNV GWAS to account for population structure. A trait-specific *P* threshold was established for each gene by permuting phenotypic values between individuals 100 times. The significance threshold was the 5% quantile (the fifth lowest *P* value from the permutations) in each set, which was then Benjamini–Hochberg-adjusted to account for multiple test bias. The effect size and variance explained by each variant was computed with FaST-LMM (v.0.6.4), with the effect sizes corresponding to the absolute value of ‘SnpWeight’, and variance corresponding to ‘EffectSize’ from the raw output. The significance thresholds were scaled to account for the different sizes of the SNP and CNV matrices. The genomic inflation factor was calculated for each trait and the *P* value was corrected when the genomic inflation factor was higher than one. To account for linkage disequilibrium among SNP or CNV loci, we grouped significant variants with an *R*^2^ > 0.6 and conflated them into a single linkage group. Within each associated linkage group, the variant with the most significant association was kept. Before filtering, 12,058 SNP-associated eQTLs and 47,082 CNV-associated eQTLs were detected as significant. After conflating the linkage groups, these numbers were reduced to 7,273 and 2,197 for SNP and CNV-associated eQTLs, respectively. For SNP-associated eQTLs, local and distant eQTLs were distinguished according to the distance from the considered gene (local eQTLs can be located 25 kb each side of the gene), all other eQTLs being considered as distant eQTLs. For CNV-associated eQTLs, we considered them local only when the variant and associated trait shared the same ORF. Significant associations can be found in datafile 4 and are summarized in Supplementary Table 9.

### Reporting summary

Further information on research design is available in the Nature Portfolio Reporting Summary linked to this article.

## Online content

Any methods, additional references, Nature Portfolio reporting summaries, source data, extended data, supplementary information, acknowledgements, peer review information; details of author contributions and competing interests; and statements of data and code availability are available at 10.1038/s41588-024-01769-9.

### Supplementary information

Supplementary Information Supplementary Figs. 1–12 and Tables 1–9.

Reporting Summary

Supplementary Tables 1–9.

## Data Availability

All sequencing reads are available at the European Nucleotide Archive under the accession no. PRJEB52153. The 1002 Yeast Genome website (http://1002genomes.u-strasbg.fr/files/RNAseq) provides access to datafile 1: normalized gene expression levels (TPM) and raw count data across 969 isolates (filename: final_data_annotated_merged_04052022.tab); datafile 2: normalized gene expression levels (TPM) and sample information for 29 replicates (file name: replicate_data_tpm_22042023.tab); datafile 3: results of allele-specific expression analyses for heterozygous introgressions and non-introgressed alleles (file name: ASE_data_counts.tab); and datafile 4: GWAS results and statistics (file name: GWAS_combined_lgcCorr_ldPruned_noBonferroni_20221207.tab).

## Code availability

All relevant scripts are available at GitHub (https://github.com/HaploTeam/1011yeastsRNAseq) and Zenodo (https://zenodo.org/records/10984278 (ref. ^56^)).
