# Supplementary material for: Pan-transcriptome reveals a large accessory genome contribution to gene expression variation in yeast
Source: Nat Genet. 2024 May 22;56(6):1278–87. doi: 10.1038/s41588-024-01769-9 (PMC11176082; doi:10.1038/s41588-024-01769-9)
Supplement: Supplementary file 1 — Supplementary Figs. 1–12 and Tables 1–9. [file 41588_2024_1769_MOESM1_ESM.pdf]

nature genetics

Supplementary information https://doi.org/10.1038/s41588-024-01769-9

Pan-transcriptome reveals a large accessory genome contribution to gene expression variation in yeast

In the format provided by the authors and unedited

This document includes:

Supplementary figures S1-12

Supplementary figure legends

Descriptions for supplementary tables S1-9

Figure S1

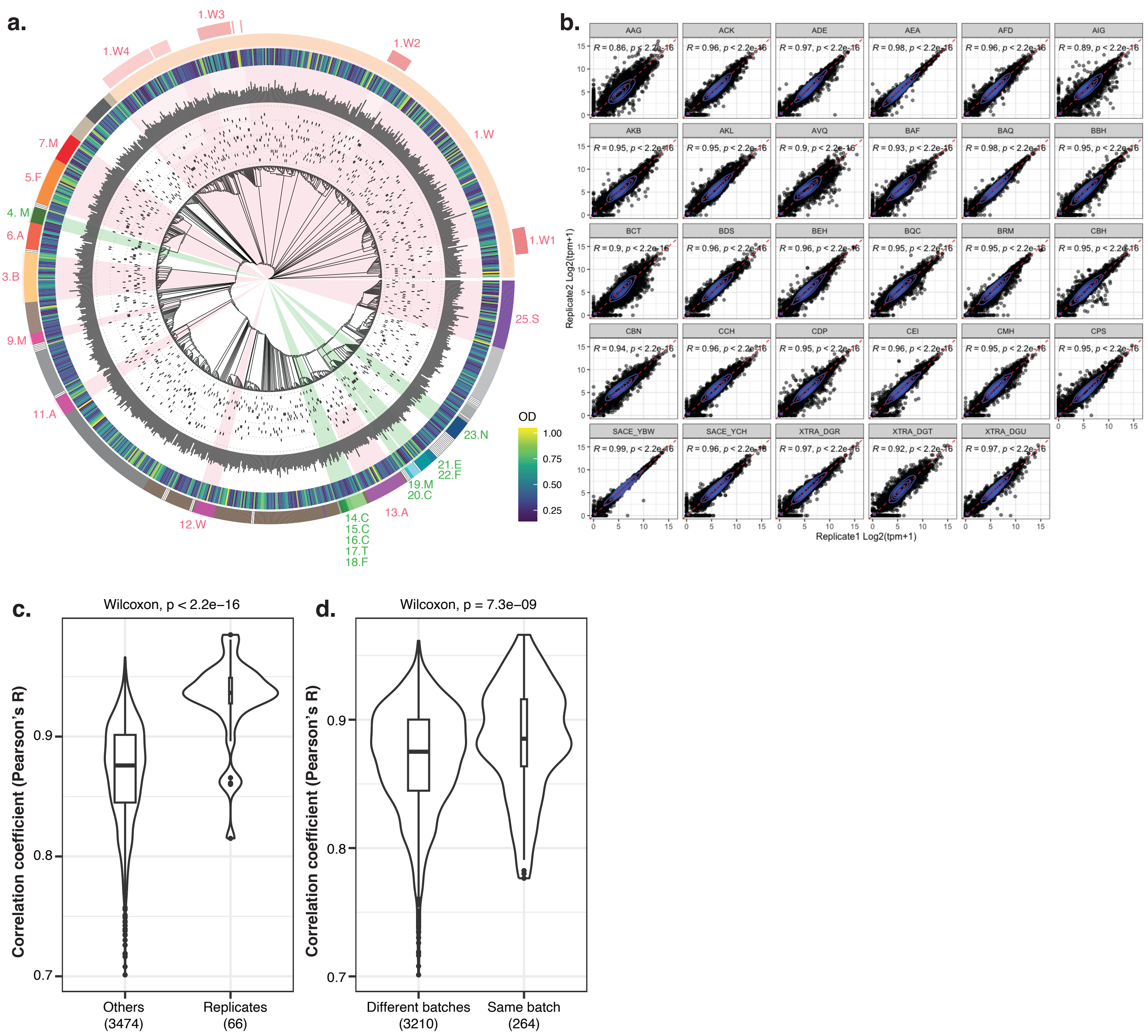

Supplemental Figure 1- Sample batch information and replicate analyses. a. From the inside to outside: circular cladogram for the 969 isolates; the library, corresponding to which 96-format plate the corresponding sample was prepared from; the total number of mapped reads for each sample; the OD value of each sample at the time of sample collection; the clades and subslades based on genomic data, cf. Figure 1. **b.** Correlation between 29 samples with replicates. Correlations were calculated based on log2(tpm+1) across 6,445 genes. Data included in online datafile 2. **c.** Distribution of pairwise-expression profile correlation between same sample replicates and across samples. **d.** Distribution of pairwise-expression profile correlation between samples within the same batch (library) and samples across different batches. Boxplot centers correspond to the median and the upper/lower bounds correspond to the the 3rd and 1st quartile. Whiskers correspond to 1.5x IQR. For both **c.** and **d.,** two-sided Wilcoxon tests were performed. Boxplot width scaled to sample sizes. Sample sizes are indicated on the x-axis label.

Figure S2

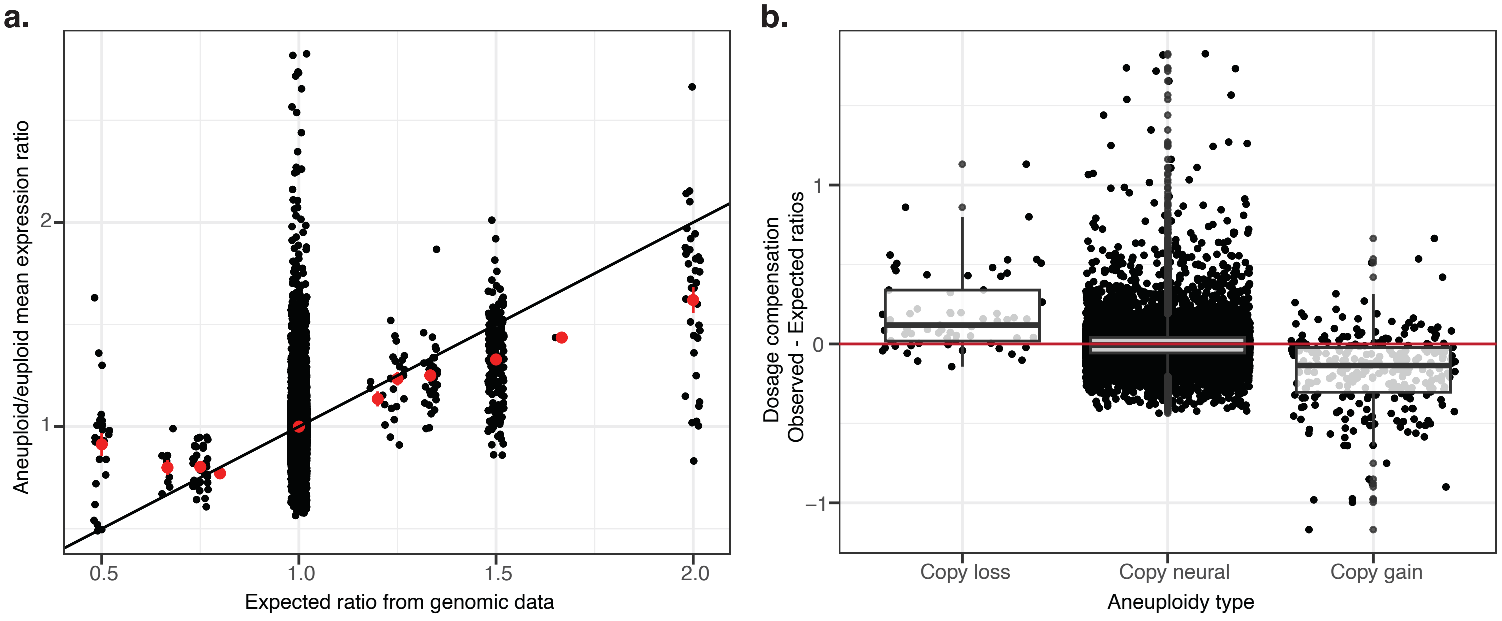

Supplemental Figure 2- Dosage compensation of aneuploid chromosomes.

a. Mean chromosomal expression level ratio between aneuploid chromosomes in a give isolate and the corresponding euploid chromosome across euploid isolates (y-axis). The expected ratios calculated from genome sequencing data are presented on the x-axis. The mean ratios and the mean standard error are indicated in red. Diagonal line indicated 1 to 1 correlation with the expected ratio. **b.** Global dosage compensation for aneuploid chromosomes. The deviation between observed and expected mean expression ratios are calculated for different types of aneuploidy (copy loss, copy neutral and copy gain). The centre of the boxplots corresponds to the median and the upper and lower bounds correspond to the 3rd and 1st quartiles. The whiskers correspond to the upper and lower bounds multiplied by 1.5 IQR. Red line indicate no compensation.

Figure S3

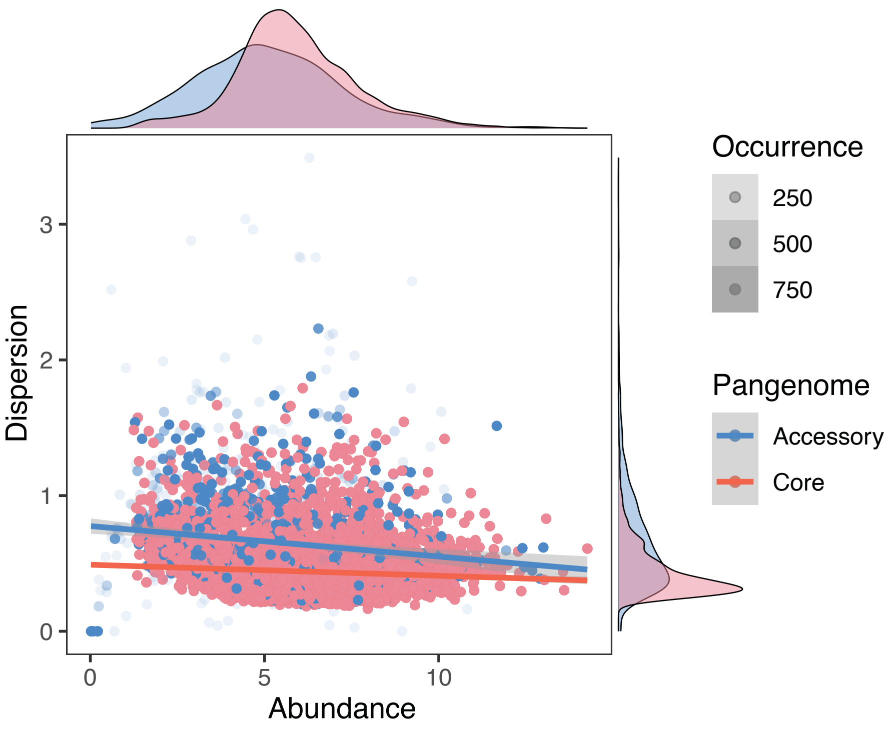

Supplemental Figure 3- Distribution of expression abundance and dispersion across core- and accessory genes. Density of the abundance and dispersion are indicated on the side. Alpha correspond to the number of occurence for genes in the accessory group. Trend lines are calculated using a linear model.

Figure S4

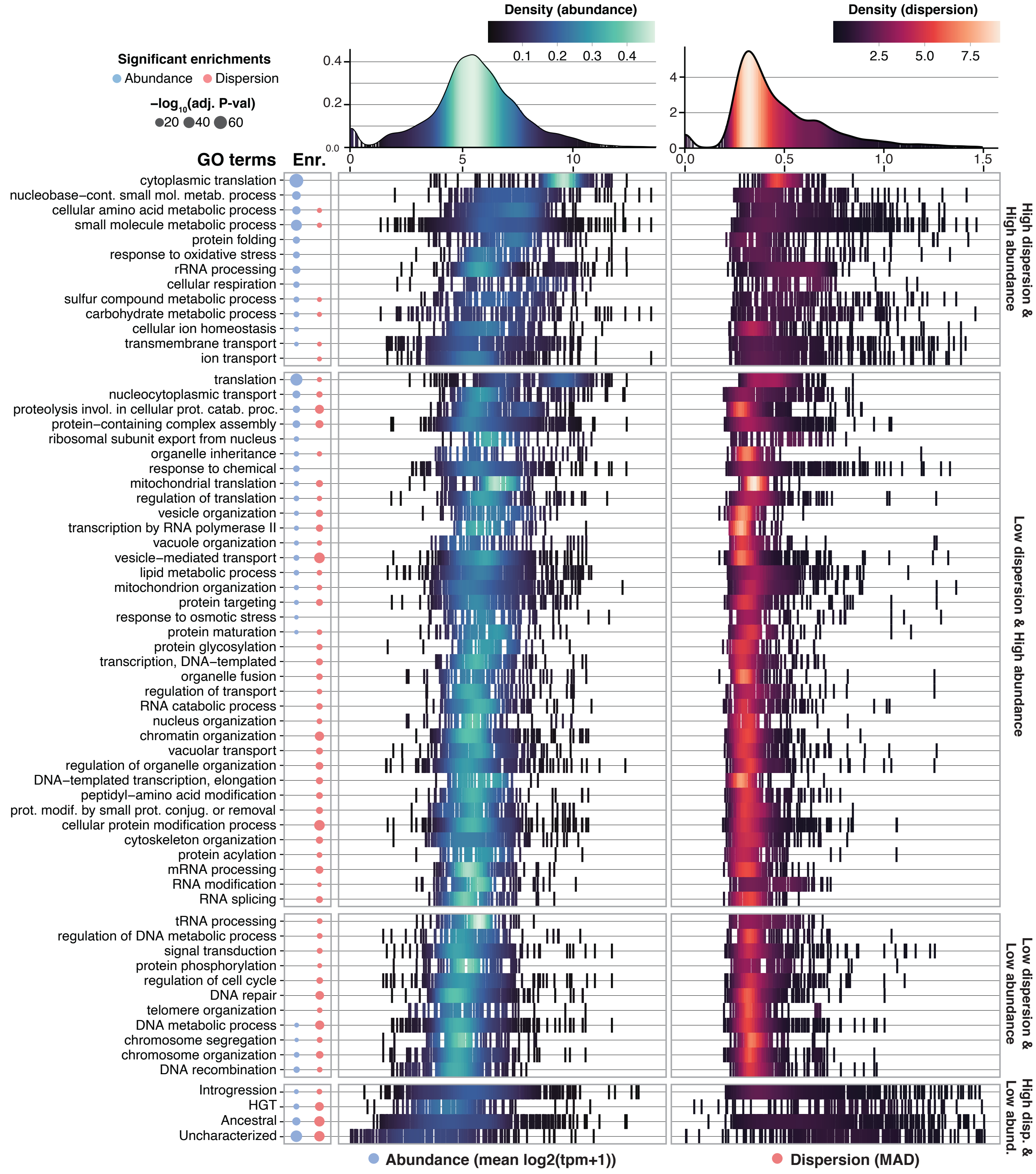

Supplemental Figure 4- Distributions and gene set enrichment analyses (GSEA) based on mean expression abundance and dispersion. The global distribution for expression abundance, calculated as the mean log2 of TPM+1, and for expression dispersion, calculated as the mean absolute deviation, are shown on the top panel. Significant BP GO slim terms (59) and accessory gene subcategories (4) are indicated on the left. The strengths of the GSEA enrichment significance are indicated using the sizes of color-coded dots. The distributions of abundance and/or dispersion values for genes assigned in the corresponding terms are plotted. The distributions are grouped according to the quadrants as shown in Figure 2d. Within each group, terms are ranked based on the median expression abundance.

Figure S5

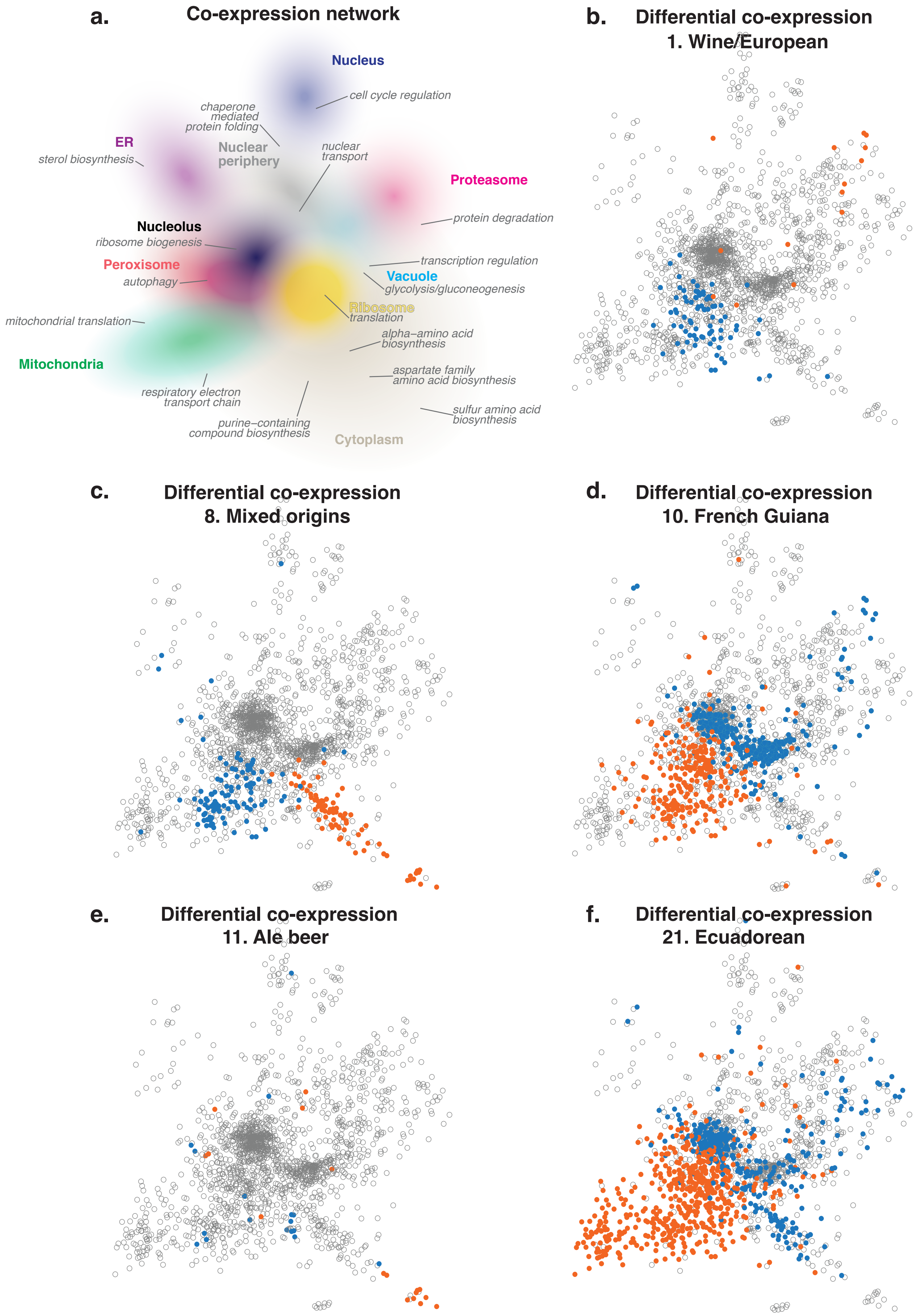
a.
Co-expression network
Nucleus
cell cycle regulation
chaperone mediated protein folding
ER
sterol biosynthesis
Nuclear periphery
nuclear transport
Proteasome
protein degradation
Nucleolus
ribosome biogenesis
Peroxisome
autophagy
transcription regulation
Vacuole
glycolysis/gluconeogenesis
Ribosome
translation
mitochondrial translation
alpha–amino acid biosynthesis
Mitochondria
aspartate family amino acid biosynthesis
respiratory electron transport chain
sulfur amino acid biosynthesis
purine–containing compound biosynthesis
Cytoplasm
b.
Differential co-expression
1. Wine/European
c.
Differential co-expression
8. Mixed origins
d.
Differential co-expression
10. French Guiana
e.
Differential co-expression
11. Ale beer
f.
Differential co-expression
21. Ecuadorean

Supplemental Figure 5- Differential co-expression by subpopulation. a. Co-expression network annotations as shown in Figure 3A. **b-f.** Significant differential co-expressions in 1. Wine/European (**b**), 8. Mixed origins (**c**), 10. French Guiana (**d**), 11. Ale beer (**e**) and 21. Ecuadorean (**f**) subpopulations. Up-regulated genes are indicated in orange and down-regulated genes in blue. Modules and genes with significant co-expression changes by subpopulations are listed in Supplemental Table 7 and recapitulated in Supplemental figure 6.

Figure S6

a.

Subpopulations	#Isolates	Differential expression* #Down (in CO)	#Up (in CO)	Diff. co-expression† Down	Up	Subpop-specific signatures Down	Up
1. Wine/European	354	310 (82)	169 (14)	M4	M5	GO:0015758	
2. Alpechin	16	143 (12)	55 (3)				
3. Brazilian bioethanol	33	182 (15)	26 (5)				GO:0019438
4. Mediterranean oak	10	56 (1)	8 (2)				
5. French dairy	30	244 (23)	188 (19)			GO:0016050	GO:0006012
6. African beer	17	175 (31)	103 (5)			GO:0016050	
7. Mosaic beer	18	18 (0)	25 (2)			GO:0055085	
8. Mixed origin	68	366(109)	187 (79)	M4	M7; M16	GO:0006772	
9. Mexican agave	7	49 (1)	20 (1)				
10. French Guiana human	30	696 (358)	604 (266)	M1; M3; M5	M2; M4; M6	GO:0016050	GO:0006772
11. Ale beer	12	118 (17)	96 (17)		M16	GO:0055085	GO:0009313
12. West African cocoa	13	35(3)	17 (4)				
13. African palm wine	26	155(4)	35 (0)			GO:0046352	
14. CHNIII	2	15 (0)	4 (0)				
15. CHNII	2	0 (0)	0 (0)				
16. CHNI	1	n.a	n.a				
17. Taiwanese	3	53 (5)	17 (1)			GO:0006739	
18. Far East Asia	9	67 (1)	9 (0)			GO:0000723	
19. Malaysian	5	97 (6)	14 (0)				
20. CHN V	2	23 (0)	8 (0)				
21. Ecuadorean	9	1007 (337)	1015 (512)	M1; M3; M5; M7	M2; M4; M6	GO:0000723	GO:0015758
22. Far East Russian	4	226 (45)	21 (4)				
23. North American oak	13	156 (8)	24 (0)				
24. Asian islands	10	36 (3)	19 (2)				
25. Sake	46	243 (34)	157 (8)			GO:0000723	GO:0006772
26. Asian fermentation	37	243 (54)	105 (10)				
M1. Mosaic region 1	17	0 (0)	4 (0)				
M2. Mosaic region 2	21	2 (0)	0 (0)				
M3. Mosaic region 3	110	18 (0)	22 (0)				
Unclustered	43	59 (4)	16 (0)			GO:0000723	

* Log2FoldChange > 0.3 & FDR < 0.05; † M1 ribosome biogenesis, M2 autophagy, M3 translation, M4 respiratory electron transport chain, M5 protein degradation, M6 mitochondrial translation, M7 alpha–amino acid biosynthesis, M16 sulfur amino acid biosynthesis.

b.

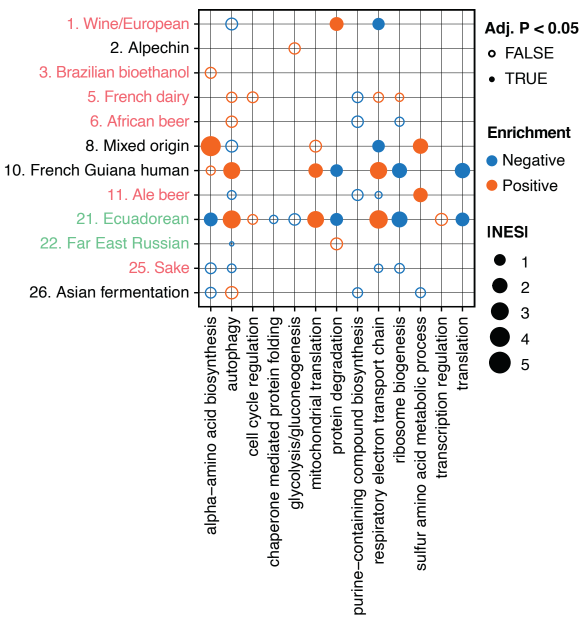

c.

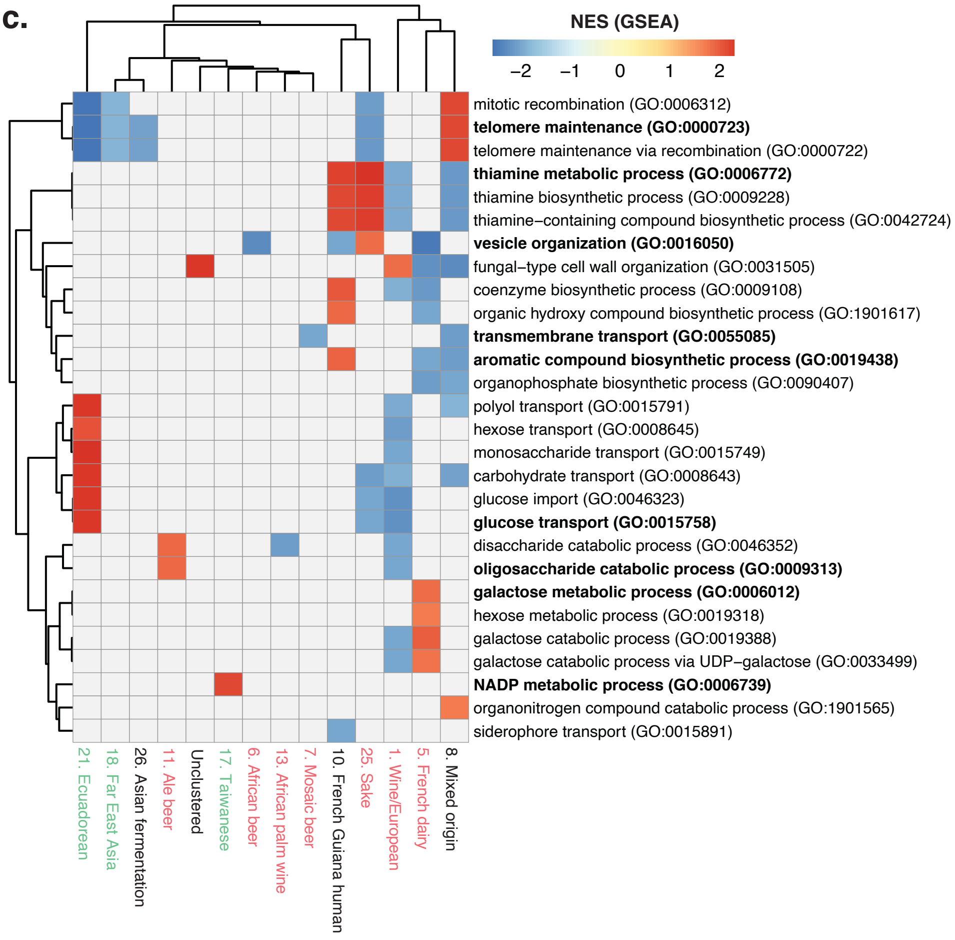

Supplemental Figure 6- Summary and functional enrichments of subpopulation specific differential (co-) expressions. a. Overview of subpopulation size, number of significant differentially expressed genes and, enriched modules and biological processes (GO terms). Bars indicate the total number of differentially expressed genes, with darker shades representing the number of differentially expressed genes that overlap with the co-expression network. Full module names are indicated at the bottom of the plot. **b.** Module overrepresentation analyses (ORA) results by subpopulation. GSEA based normalized enrichment scores (NES) are shown. Dot sizes represent the absolute NES, with blue dots corresponding to underrepresentation (negative) and orange corresponding to overrepresented genes (positive). Solid dots indicate significant scores with adjusted P value < 0.05 (FDR) based 10,000 permutations. **c.** GSEA on log2 fold-change ranked differentially expressed genes by subpopulations. The heatmap is clustered based on the normalized enrichment scores across subpopulations and biological processes GO terms. Subpopulations with no enrichments are not shown. NES with an adjusted P-value > 0.05 (FDR) are masked in grey. FDR estimated based on 10,000 permutations.

Figure S7

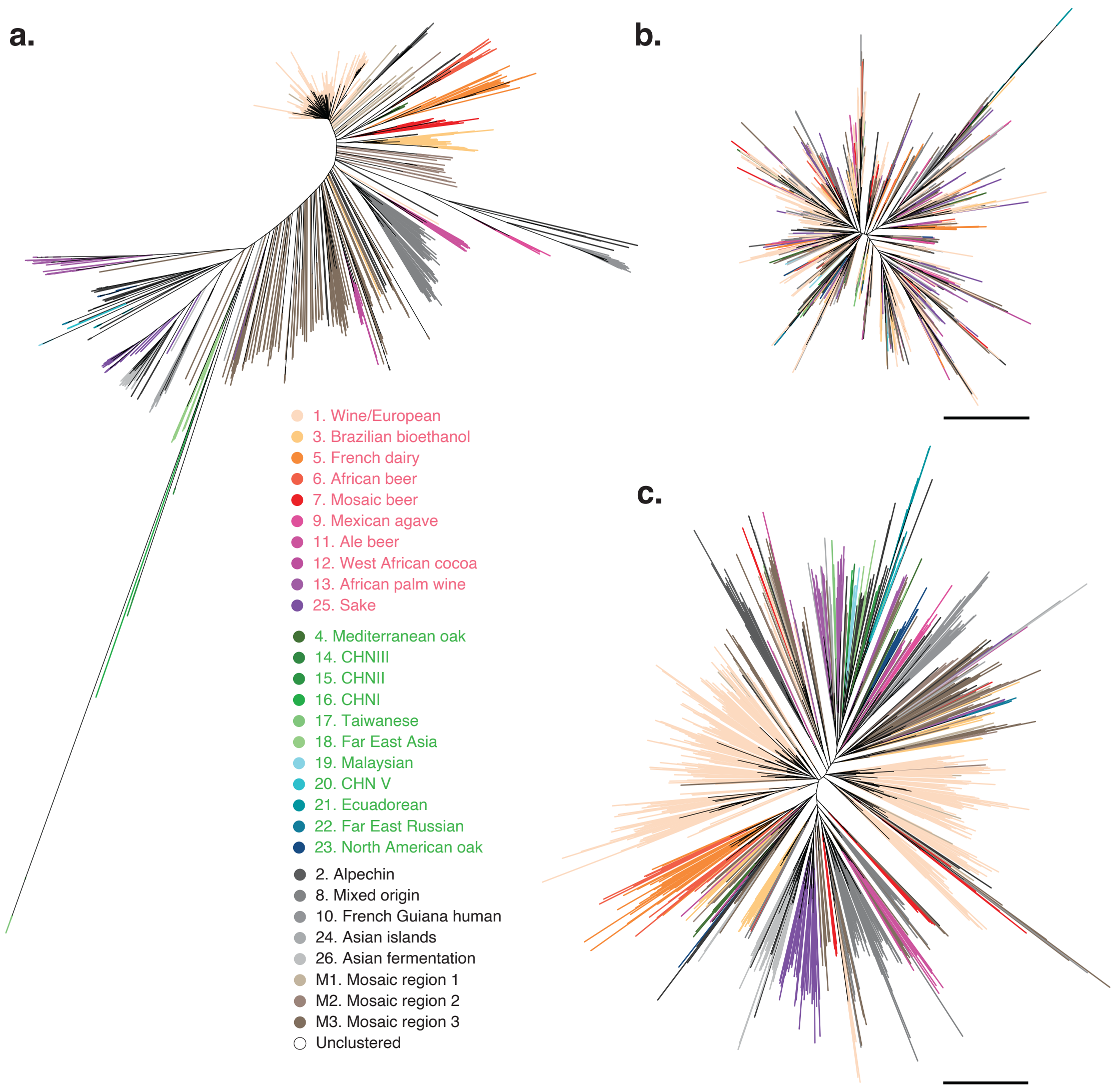

Supplemental Figure 7- Genetic divergence based on SNPs vs. gene expression diversity.
a. Neighbor-joining tree based on biallelic SNP positions across 969 isolates. Branches are color-coded according to the subpopulation definition as shown in Figure 1a. **b-c.** Neighbor-joining trees based on Euclidean distances using the 1,797 co-expression genes (**b**) and 2,209 differential expression genes (**c**). Scale bars are as indicated.

Figure S8

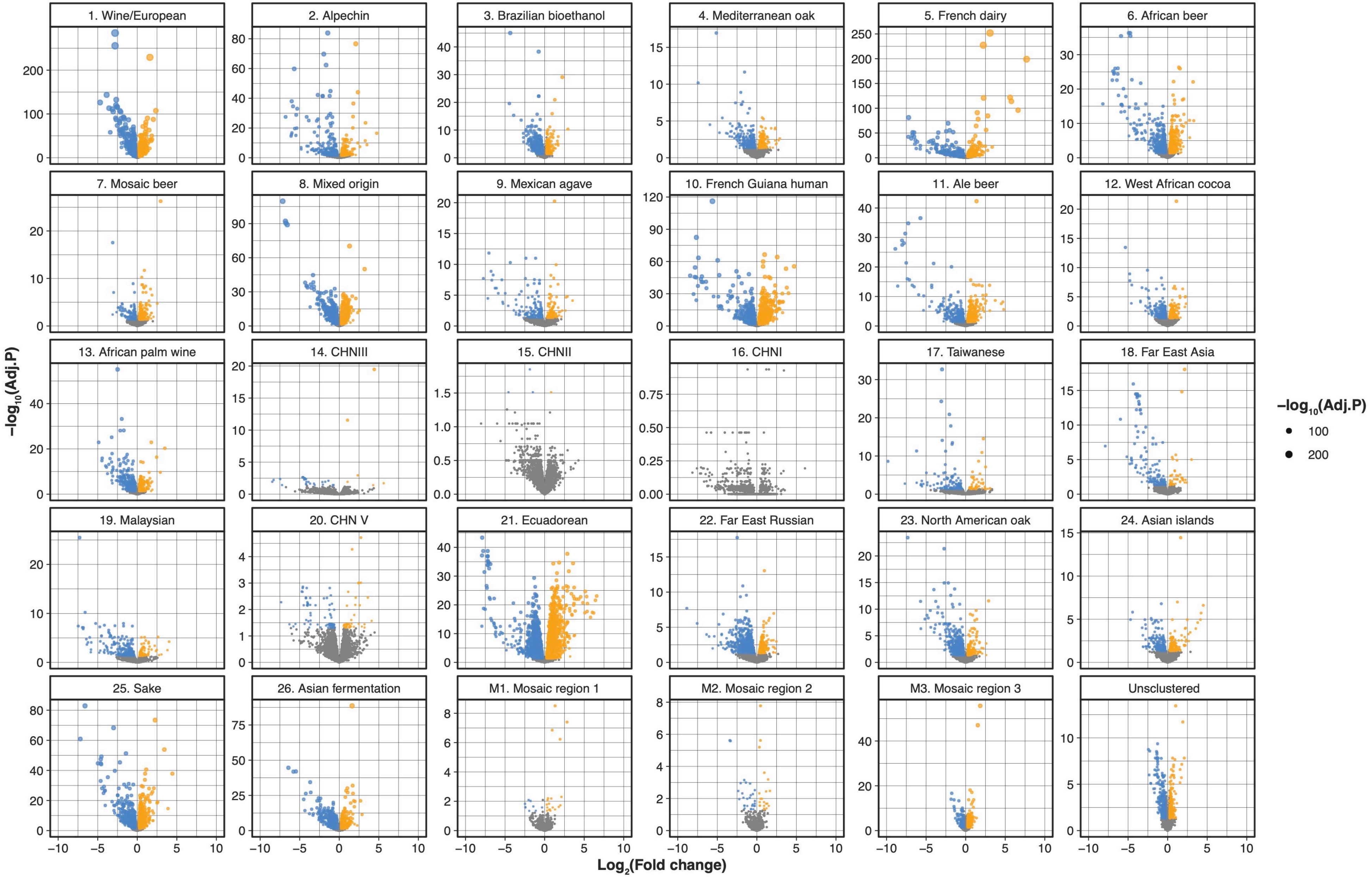
1. Wine/European
2. Alpechin
3. Brazilian bioethanol
4. Mediterranean oak
5. French dairy
6. African beer
7. Mosaic beer
8. Mixed origin
9. Mexican agave
10. French Guiana human
11. Ale beer
12. West African cocoa
13. African palm wine
14. CHNIII
15. CHNII
16. CHNI
17. Taiwanese
18. Far East Asia
19. Malaysian
20. CHN V
21. Ecuadorean
22. Far East Russian
23. North American oak
24. Asian islands
25. Sake
26. Asian fermentation
M1. Mosaic region 1
M2. Mosaic region 2
M3. Mosaic region 3
Unsclustered
$-\log_{10}$(Adj.P)
$\log_2$(Fold change)
$-\log_{10}$(Adj.P)
100
200

Supplemental Figure 8- Volcano plots for differential gene expressions detected across each subpopulation. Subpopulations are indicated on each subplot. For each subplot, x-axis corresponds to the log2 fold-change for each gene and y-axis correspond to the -log10 of the B-H adjusted P-values. Genes with an adjusted P-value < 0.05 and an absolute log2 foldchange > 0.3 are colored in blue for down-regulated genes and orange for up-regulated genes. Dot sizes are scaled to the y-axis.

Figure S9

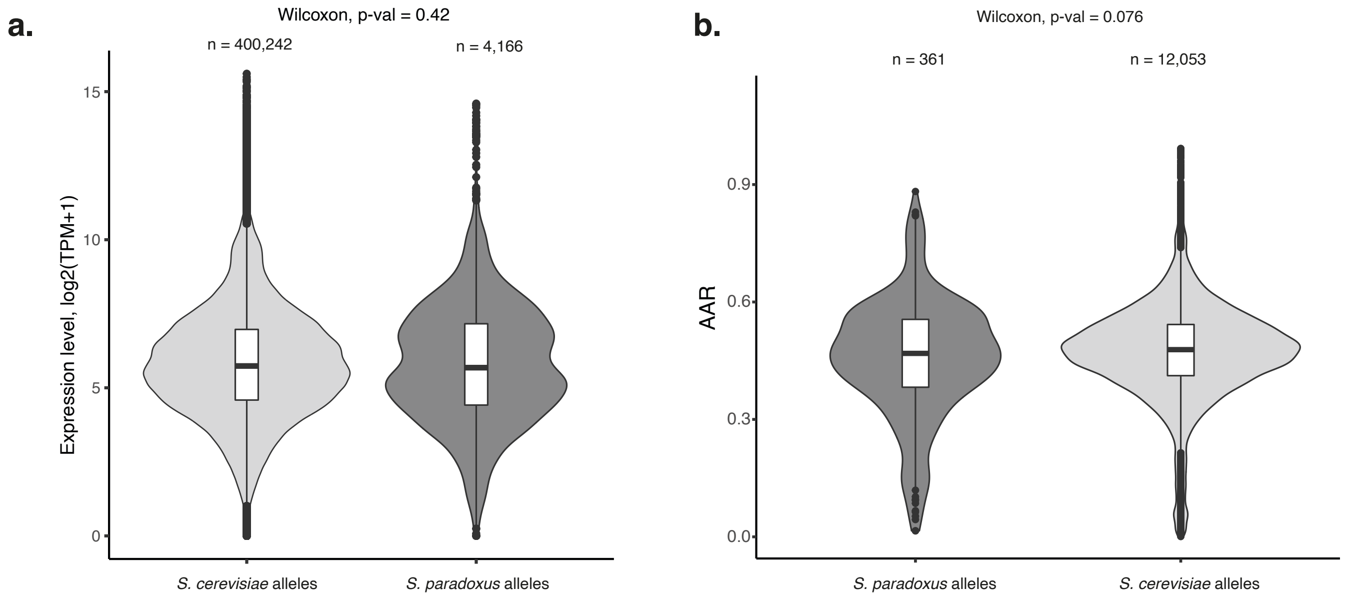

Supplemental Figure 9- Comparison of *S. cerevisiae* and *S. paradoxus* allele expression. **a.** Gene expression level comparison between 437 genes homozygous either for *S. cerevisiae* or *S. paradoxus* allele. The p-value is calculated using a two-sided Wilcoxon test. Sample sizes are indicated on the plot. **b.** Comparison between the alternative allele ratio (AAR) from heterozygous introgressed sites (after genetic allele balance filtration) and the AAR from non-introgressed heterozygous sites. The p-value is calculated using a two-sided Wilcoxon test. Sample sizes are indicated on the plot. All boxplot centres correspond to the median and the upper and lower bounds correspond to the 3rd and 1st quartiles. The whiskers correspond to the upper and lower bounds time 1.5 IQR.

Figure S10

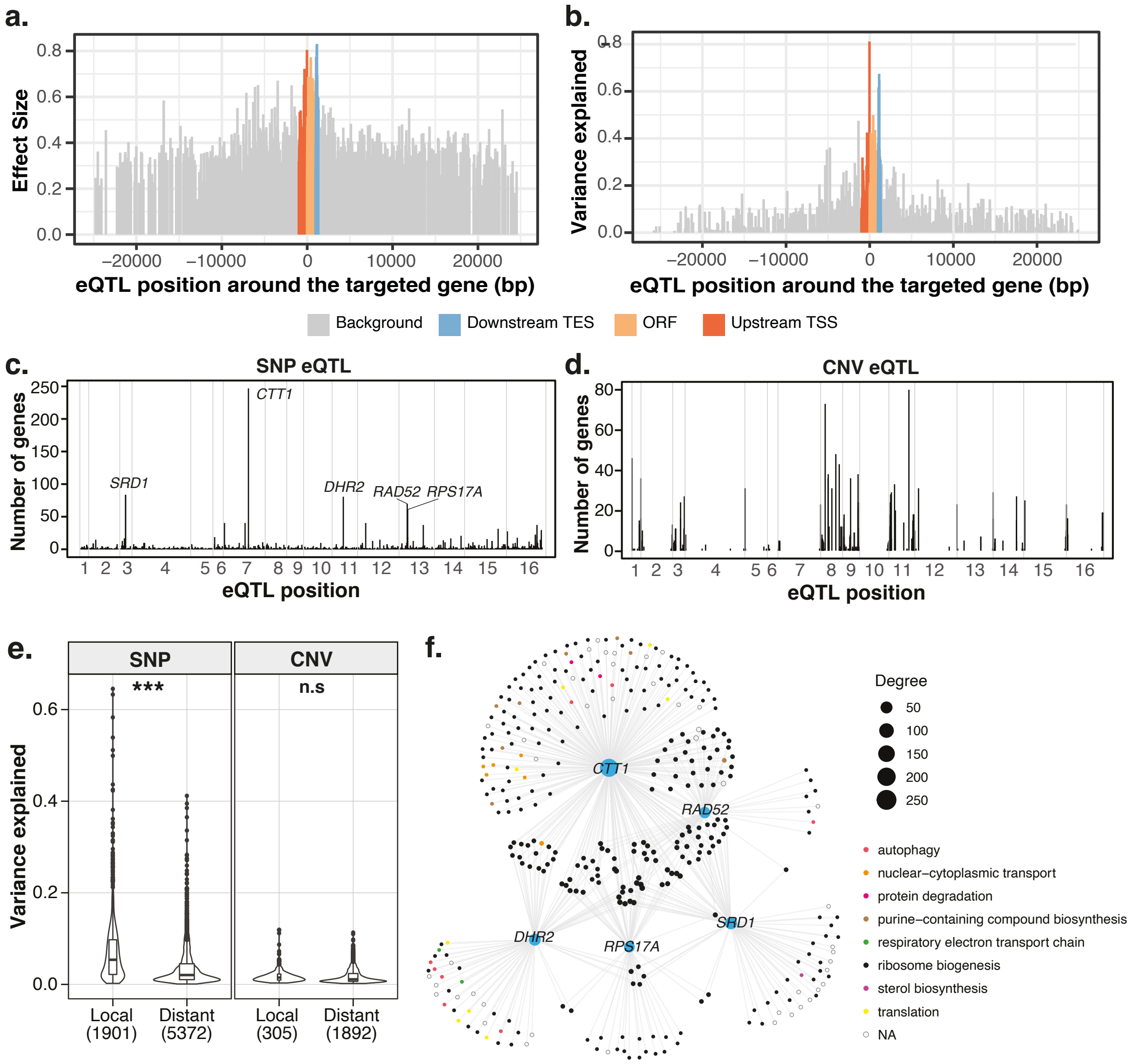

Supplemental Figure 10- eQTL feature summaries.
a-b. Relative location of the local eQTL around the targeted gene and their effect sizes (**a**) and variance explained (**b**). The upstream region (red) corresponds to the 1,000 bp before the transcription start site (TSS), the downstream region (blue) corresponds to the 300 bp after the stop codon. The position of the eQTL located within the ORF (yellow) is scaled to 1,000 bp. The background regions contain the remaining regions located 25 kb before and after the gene. **c.** Hotspots for trans SNP-eQTL. The x-axis indicates the chromosomal locations for the eQTL and y-axis corresponds to the number of gene expression traits that are associated with a given eQTL. **d.** Hotspots for trans CNV-eQTL. The x-axis indicates the chromosomal locations for the eQTL and y-axis corresponds to the number of gene expression traits that are associated with a given eQTL. **e.** Comparison of the fraction of variance explained between local and distant eQTL for SNP and CNV eQTL types. Significant differences are indicated with stars. Sample sizes are indicated on the x-axis labels. P-value derived from two-sided Wilcoxon test. P-value for local vs distant SNP-eQTL <2.2e-16. P-value for local vs distant CNV-eQTL =0.15. Boxplot centers correspond to the median and upper/lower bounds correspond to the 3rd and 1st quartile. Whiskers extends to 1.5 times IQR. **f.** eQTL association network for top hotspots involving more than 50 expression traits. Size correspond to interaction degrees and expression genes are color-coded according to co-expression modules.

Figure S11

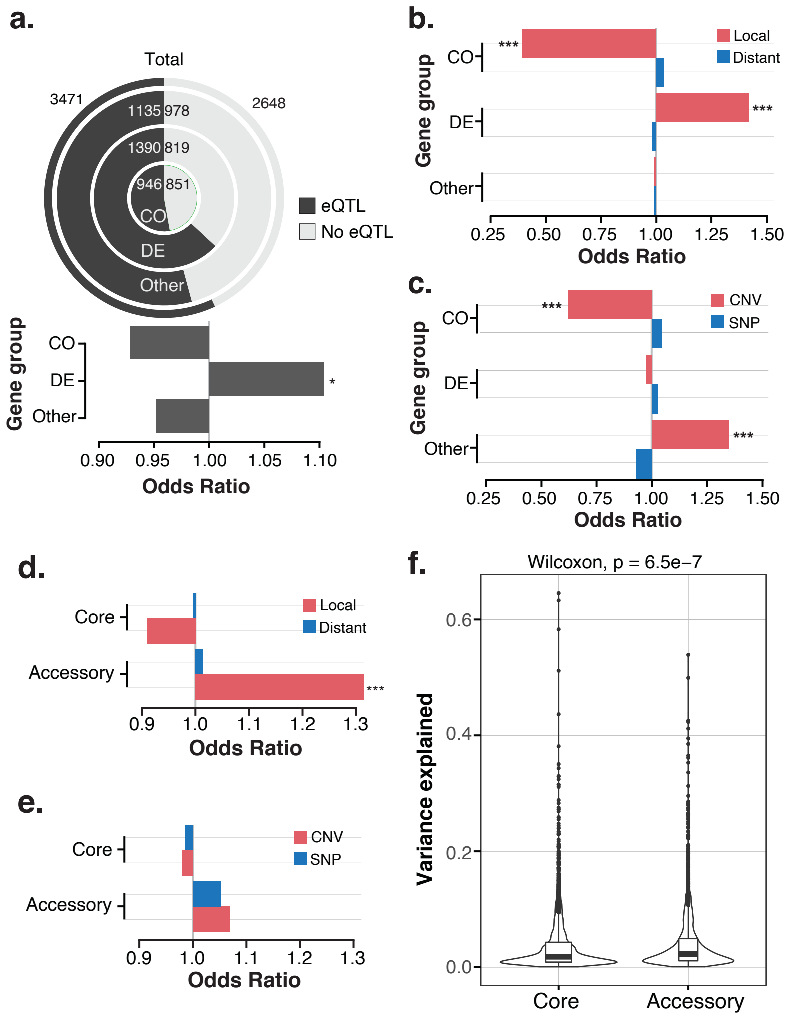

Supplemental Figure 11- eQTL enrichments across different transcriptional network levels.
a. From the inside out: the number and proportion of genes that are impacted by at least one eQTL for co-expression genes (CO), differential expression genes (DE), the remaining traits (Other) and the total dataset (outer ring). Fold-en- richments for CO, DE and Other genes on the proportion of genes impacted by at least one eQTL compared to the total set are presented as bars (odds ratio). Significant enrichment or depletion based on two-sided Fisher tests is indicated with stars. **b.** Fold-enrichments for CO, DE and Other genes on the proportion of genes impacted by at least one local (red) or distant (blue) eQTL com- pared to the total set. Odds ratios are indicated on the x-axis based on two-sided Fisher test. **c.** Fold-en- richments for CO, DE and Other genes on the proportion of genes impacted by at least one SNP (red) or CNV (blue) eQTL com- pared to the total set. Odds ratios are indicated on the x-axis based on two-sided Fisher test. **d.** Fold-enrichments for core and accessory genes on the proportion of genes impacted by at least one local (red) or distant (blue) eQTL compared to the total set. Odds ratios are indicated on the x-axis based on two-sided Fisher test. **e.** Fold-enrichments core and accessory genes on the proportion of genes impacted by at least one CNV (red) or SNP (blue) eQTL compared to the total set. Odds ratios are indicated on the x-axis based on two-sided Fisher test. **f.** Comparison of the fraction of variance explained between eQTL for accessory and core gene categories. Boxplot centers correspond to the median and upper/lower bounds correspond to the 3rd and 1st quartile. Whiskers extends to 1.5 times IQR. Two-sided Wilcoxon test p-value is indicated. Sample size N = 3,378 for accessory genes and N = 6,092 for core genes.

Figure S12

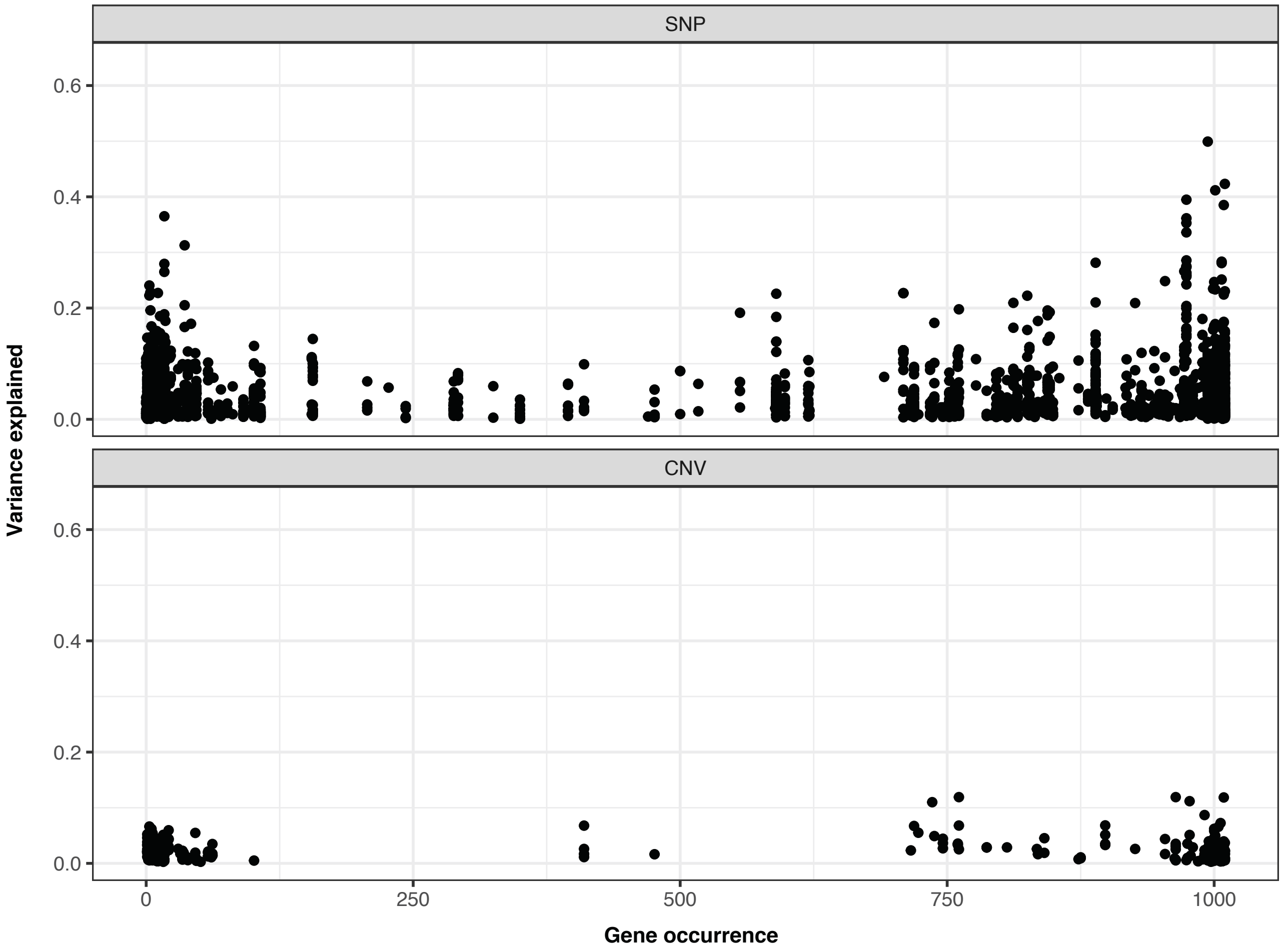

Supplemental Figure 12- Distribution of the fraction of variance explained per eQTL across accessory genes. For each accessory gene associated with at least on eQTL, the fraction of variance explained (y-axis) is plotted against the number of isolates that carry the given accessory gene, i.e. gene occurrence, on the x-axis. The gene occurrence was obtained from the pangenome annotations across 1,011 isolates (Peter et al. 2018).

Supplementary table descriptions

Table S1- Description of isolates included in this study.

Table S2- Description of genes included in this study.

Table S3- GSEA results on gene expression abundance and dispersion.

Table S4- Co-expression network module definition and connectivity

Table S5- GO term enrichment results across co-expression modules. This table contains two tabs:

- BP: enrichments across GO biological processes;
- CC: enrichments across GO cellular compartments.

Table S6- Significant differential gene expression across subpopulations.

Table S7- Differential co-expression by subpopulation based on module overrepresentation analyses

Table S8- GSEA results on differential gene expression by subpopulation

Table S9- eQTL enrichment analyses across transcriptional network levels. This table contains three tabs:

- counts: summary counts for the types of eQTL associated with a given gene expression trait. Expression traits are grouped into co-expression (CO), differential expression (DE) and the remaining cases (Other).
- Fisher's test summary statistics for CO, DE and Other genes
- Fisher's test summary statistics for core and accessory genes
