# Supplementary material for: Pan-transcriptome reveals a large accessory genome contribution to gene expression variation in yeast
Source: Nat Genet. 2024 May 22;56(6):1278–87. doi: 10.1038/s41588-024-01769-9 (PMC11176082; doi:10.1038/s41588-024-01769-9)
Supplement: Supplementary file 2 — Reporting Summary [file 41588_2024_1769_MOESM2_ESM.pdf]

nature portfolio

Corresponding author(s): Jing Hou and Joseph Schacherer

Last updated by author(s): 03/25/2024



# Reporting Summary

Nature Portfolio wishes to improve the reproducibility of the work that we publish. This form provides structure for consistency and transparency in reporting. For further information on Nature Portfolio policies, see our Editorial Policies and the Editorial Policy Checklist.

## Statistics

For all statistical analyses, confirm that the following items are present in the figure legend, table legend, main text, or Methods section.

| n/a | Confirmed | |
|---|---|---|
| ☐ | ☒ | The exact sample size (*n*) for each experimental group/condition, given as a discrete number and unit of measurement |
| ☐ | ☒ | A statement on whether measurements were taken from distinct samples or whether the same sample was measured repeatedly |
| ☐ | ☒ | The statistical test(s) used AND whether they are one- or two-sided<br>*Only common tests should be described solely by name; describe more complex techniques in the Methods section.* |
| ☒ | ☐ | A description of all covariates tested |
| ☐ | ☒ | A description of any assumptions or corrections, such as tests of normality and adjustment for multiple comparisons |
| ☐ | ☒ | A full description of the statistical parameters including central tendency (e.g. means) or other basic estimates (e.g. regression coefficient) AND variation (e.g. standard deviation) or associated estimates of uncertainty (e.g. confidence intervals) |
| ☐ | ☒ | For null hypothesis testing, the test statistic (e.g. *F*, *t*, *r*) with confidence intervals, effect sizes, degrees of freedom and *P* value noted<br>*Give P values as exact values whenever suitable.* |
| ☒ | ☐ | For Bayesian analysis, information on the choice of priors and Markov chain Monte Carlo settings |
| ☒ | ☐ | For hierarchical and complex designs, identification of the appropriate level for tests and full reporting of outcomes |
| ☐ | ☒ | Estimates of effect sizes (e.g. Cohen's *d*, Pearson's *r*), indicating how they were calculated |

*Our web collection on statistics for biologists contains articles on many of the points above.*

## Software and code

Policy information about availability of computer code

| | |
|---|---|
| Data collection | Raw reads were cleaned with cutadapt; mapping of cleaned reads was performed using TopHat (v2.0.13); The resulting bam files were sorted and indexed using SAMtools (v1.9). Duplicated reads were marked using Picard (v2.18.14) in GATK (v4.1.0.0). HaplotypeCaller (command in GATK) was used to call variants in each individual sample. Mapping and ORF annotations were performed using STAR (2.5.2b). Mapped read counts were obtained using the featureCounts function from the Subread (2.0.2) package in R. |
| Data analysis | All analyses were performed using R (4.2.1). The following packages were used: ape (5.6-2), SNPrelate (1.32.2), DEseq2 (1.38.3), sna (2.7-1), WGCNA (1.72-1), fastcluster (1.2.3), dynamicTreeCut (1.63-1), CEMitool (1.22.0), fgsea (1.24.0), rrvgo (1.10.0). GWAS was performed using FaST-LMM (0.6.4). Codes available at https://github.com/HaploTeam/1011yeastsRNAseq. |

For manuscripts utilizing custom algorithms or software that are central to the research but not yet described in published literature, software must be made available to editors and reviewers. We strongly encourage code deposition in a community repository (e.g. GitHub). See the Nature Portfolio guidelines for submitting code & software for further information.

## Data

Policy information about availability of data

All manuscripts must include a data availability statement. This statement should provide the following information, where applicable:

- Accession codes, unique identifiers, or web links for publicly available datasets
- A description of any restrictions on data availability
- For clinical datasets or third party data, please ensure that the statement adheres to our policy

All sequencing reads are available in the European Nucleotide Archive (ENA) under the accession number PRJEB52153.
The 1002 Yeast Genome website - http://1002genomes.u-strasbg.fr/files/RNAseq provides access to:
- Datafile S1: final_data_annotated_merged_04052022.tab
- Datafile S2: replicate_data_tpm_22042023.tab
- Datafile S3: GWAS_combined_lgcCorr_ldPruned_noBonferroni_20221207.tab

## Human research participants

Policy information about studies involving human research participants and Sex and Gender in Research.

| | |
|---|---|
| Reporting on sex and gender | not applicable |
| Population characteristics | not applicable |
| Recruitment | not applicable |
| Ethics oversight | not applicable |

Note that full information on the approval of the study protocol must also be provided in the manuscript.

# Field-specific reporting

Please select the one below that is the best fit for your research. If you are not sure, read the appropriate sections before making your selection.

☒ Life sciences ☐ Behavioural & social sciences ☐ Ecological, evolutionary & environmental sciences

For a reference copy of the document with all sections, see nature.com/documents/nr-reporting-summary-flat.pdf

# Life sciences study design

All studies must disclose on these points even when the disclosure is negative.

| | |
|---|---|
| Sample size | The same samples from the 1,011 yeast natural isolate collection (Peter et al. 2018, Nature) were used, which is the largest set currently available in this species. Statistical power for GWAS using the same population was previously established in Peter et al. 2018 for growth phenotypes. |
| Data exclusions | A total of 1,046 samples were sequenced, then ranked based on the number of shared rare SNPs with each relevant strain described in the SNP matrix. This allows to automatically validate 940 unique isolates for which the expected strain was among the top 3 ranking strains. The remaining samples were manually investigated: 24 samples that were part of a large cluster of closely related strains could be validated as the expected strain and 19 samples could be unambiguously reassigned to the top 1 ranking strain. 14 samples out of the 1,046 could not be validated or reassigned and were discarded from the remaining analyses. A total of 18 samples were further excluded with less than 1 million mapped reads. After this step, a final set of 969 unique isolates was retained. |
| Replication | Biological replicates were done for 29 samples to check for reproducibility. All replicates data were successfully obtained. |
| Randomization | The strains were grouped in 96-well plates according to their growth rates and then grown in 1 ml of liquid SC media using deep well blocks until reaching the mid-log phase. No correlation between growth and subpopulations were observed. |
| Blinding | All analyses were performed without preconception of the ecological and geographical origins, domestication status or genetic similarity between the samples. |

# Reporting for specific materials, systems and methods

We require information from authors about some types of materials, experimental systems and methods used in many studies. Here, indicate whether each material, system or method listed is relevant to your study. If you are not sure if a list item applies to your research, read the appropriate section before selecting a response.

## Materials & experimental systems

| n/a | Involved in the study |
| --- | --- |
| ☒ | ☐ Antibodies |
| ☒ | ☐ Eukaryotic cell lines |
| ☒ | ☐ Palaeontology and archaeology |
| ☒ | ☐ Animals and other organisms |
| ☒ | ☐ Clinical data |
| ☒ | ☐ Dual use research of concern |

## Methods

| n/a | Involved in the study |
| --- | --- |
| ☒ | ☐ ChIP-seq |
| ☒ | ☐ Flow cytometry |
| ☒ | ☐ MRI-based neuroimaging |
